# Design, synthesis, biological evaluation and computational studies of novel phthalimides as dual COX-2/5-LOX inhibitors

**DOI:** 10.1007/s11030-025-11431-z

**Published:** 2026-01-21

**Authors:** Rasha M. Hassan, Mohammed S. Abdel-Maksoud, May A. El-Manawaty, Ibrahim M. Ibrahim, Walaa H. Abd-Allah, Aida A. El-Azzouny, Mohamed N. Aboul-Enein

**Affiliations:** 1 Medicinal and Pharmaceutical Chemistry Department, Pharmaceutical and Drug Industries Research Institute, National Research Centre (ID: 60014618), P.O. 12622, Giza Dokki, Egypt; 2 Pharmacognosy Department, National Research Centre (ID: 60014618), 12622, Dokki, Giza Egypt; 3 Biophysics Department, Faculty of Science, Cairo University, Cairo, Giza 12613 Egypt; 4 Pharmaceutical Chemistry Department, Collage of Pharmaceutical Science and Drug Manufacturing, Misr University for Science and Technology, P.O. 77, 6th of October City, Giza Egypt

**Keywords:** Phthalimides, COX-2, 5-LOX, RAW 264.7 macrophages.

## Abstract

Aiming to find effective anti-inflammatory agents, two new series of phthalimide derivatives **4a-h** and **7a-f** were synthesized and evaluated as inhibitors of COX and 5-LOX enzymes. The structures of all of the new compounds were confirmed by different spectroscopic analyses. The compounds in this study were designed with the intention of being moderately selective COX-2 inhibitors to avoid the negative effects of both highly selective COX-2 and non-selective COX inhibitors. The results of the in vitro screening showed that compounds **4c** and **7a** are the most potent compounds on COX-2 isoenzyme with moderate selectivity indices lower than that of celecoxib but higher than meloxicam. The two compounds also demonstrated powerful 5-LOX inhibitory activity with IC_50_ values less than 1 µM which is comparable to that of the reference drug zileuton. In addition all of the compounds under investigation could inhibit the nitric oxide production in LPS-induced RAW 264.7 macrophages. Furthermore, compounds **4c** and **7a** caused a reduction of the inflammatory mediators PGE-2, IL-6 and TNF-α supporting their anti-inflammatory effect in macrophages. Finally, molecular docking studies as well molecular dynamic simulations were carried out to investigate the binding pattern of the most potent compounds into the active site of both COX-2 and 5-LOX. Consequently, the achieved findings of this study suggest that those novel phthalimide derivatives could be promising leads for further optimization and the development of new anti-inflammatory drugs.

**Graphical abstract:**

**Figure:**
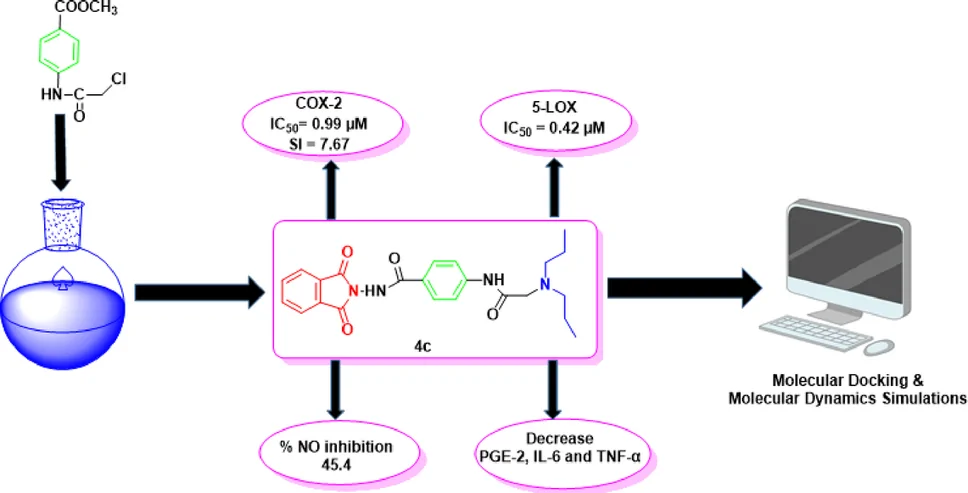

New phthalimide derivatives were synthesized as anti-inflammatory agents. Compound **4c** inhibited both COX-2 and LOX enzymes and production of NO. Also, it decreased the levels of PGE-2, IL-6 and TNF-α.

**Supplementary Information:**

The online version contains supplementary material available at https://doi.org/10.1007/s11030-025-11431-z.

## Introduction

Inflammation is considered as a defense mechanism in the body tissues that originates due to stimuli of infection, injuries or toxic substances. Improper control of inflammation may lead to chronic or serious systemic inflammatory diseases [1, 2]. Upon extracellular stimulation, macrophages produce pro-inflammatory mediators, including prostaglandin E2 (PGE-2), nitric oxide (NO), interleukins (IL-1), tumor necrosis factor (TNF-α) as well as other inflammatory mediators [3]. Non-steroidal anti-inflammatory drugs (NSAIDs) are a widely used class to control inflammatory diseases [4]. NSAIDs exert their anti-inflammatory activity by inhibition of cyclooxygenase (COX), which is responsible for the synthesis of the inflammatory mediators, including prostaglandins, prostacyclin and thromboxanes from arachidonic acid [5]. There are two major isoforms of COX enzyme, the constitutive COX-1 which has an important function in the regulation of the renal homeostasis, platelets aggregation and protection of the gastric mucosa. Meanwhile, COX-2 is inducible and it is produced in the tissue as a reaction to the inflammation to generate inflammatory mediators [6–8]. Accordingly, suppression of COX-2 leads to the required therapeutic effects of NSAIDs. The use of non-selective NSAIDs that target both COX-1 and COX-2 is accompanied by serious side effects as gastrointestinal ulceration and problems in kidney [9]. Therefore, efforts have been directed towards the development of selective COX-2 inhibitors such as celecoxib that are free from the previous unwanted side effects. However, the long term use of the latter class of drugs is linked with cardiovascular thrombotic events and myocardial infarction that led to withdrawal of rofecoxib and valdecoxib from the market [10, 11]. These side effects on the cardiovascular system are due to the selective COX-2 inhibition that leads to a strong decrease in the levels of prostacyclin (PGI2) which has vasodilatory and anti-aggregatory effects without a synchronous change in the levels of the platelet COX-1-derived prothrombotic thromboxane A2. Consequently, PGI2 production suppression removed the protective limitation on thrombogenesis, atherogenesis and hypertension [12, 13]. Therefore, the search for new safe NSAIDs that lack the side effects of both non-selective COX inhibitors and highly selective COX-2 inhibitors is an urgent need. The new strategy in the development of selective COX-2 inhibitors is to keep a slight effect on COX-1 to limit the prostacyclin/thromboxane A2 imbalance and decrease the potential of cardiovascular side effects [6, 14]. Aiming to achieve optimum safety margin the new an anti-inflammatory agent should has COX-1/COX-2 ratio less than that of celecoxib and higher than meloxicam, that is to say the drug should be selective but not specific to COX-2 [13, 15].

Studying the structures of both COX-1 and COX-2 revealed that COX-2 active site is more wider by about 20% than COX-1. This is due to the replacement of the bulky isoleucine amino acid at positions 434 and 532 in COX-1 with smaller valine residues in COX-2. This led to formation of an additional secondary pocket in COX-2 that has a hydrophilic nature and responsible for selectivity of COX-2 inhibitors. The rest of the interactions attained by the selective COX-2 inhibitors are hydrophobic in nature with the hydrophobic areas in COX active site [4].

**Fig. 1:**
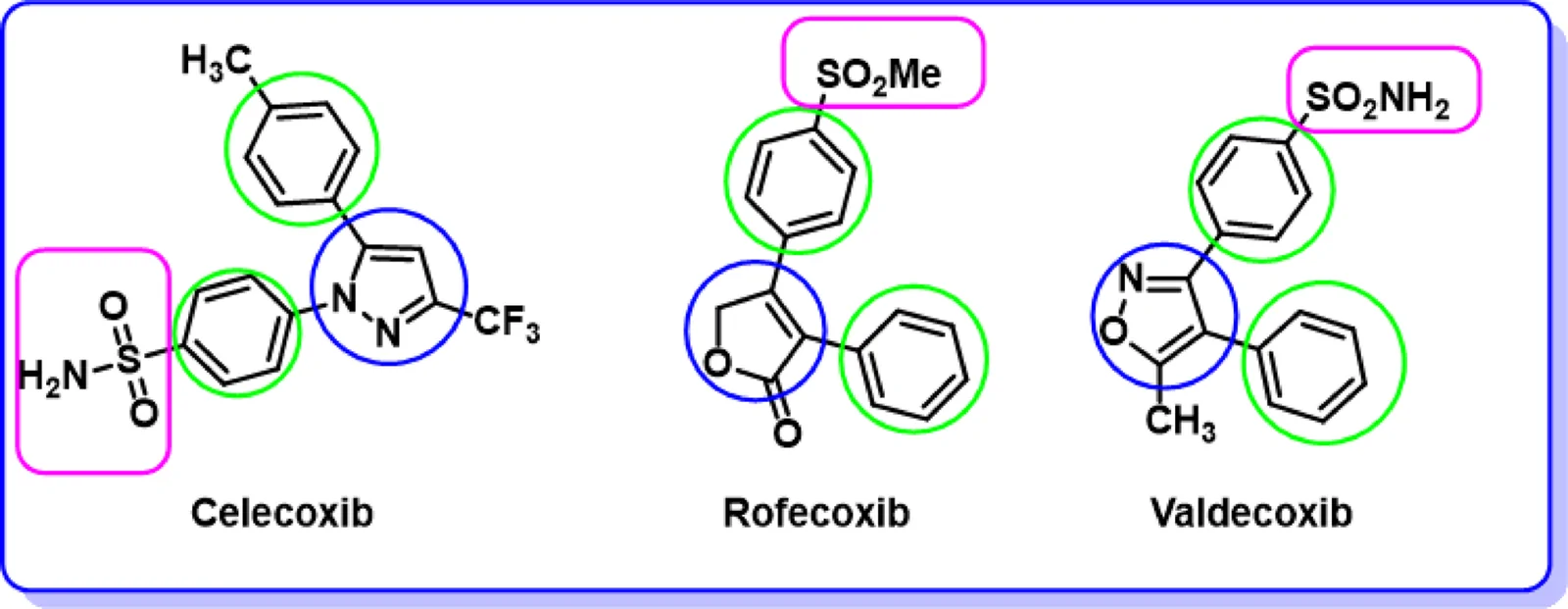
Structures of selective COX-2 inhibitor drugs with illustration of the pharmacophore features

Inspite of the presence of various chemical structures of the reported COX-2 inhibitors, the structural activity relationship studies identified that they usually have a central carbocyclic or heterocyclic core substituted with diaryl moieties and one of them has a sulfonamide or sulfone groups at the para position (Fig. 1) [14, 16, 17]. The latter groups are the reason of the high selectivity towards COX-2 isoenzyme because of their capability to make hydrogen bonds with the hydrophilic amino acid residues (His90, Gln192, Arg513) located at the COX-2 specific side pocket [18–20]. Nevertheless, in many reported selective COX-2 the sulfonyl is not found [6, 14, 21, 22].

As a result of suppression of COX enzymes the metabolism of arachidonic acid shifts towards the 5-lipoxygenase (5-LOX) pathway leading to increase in leukotrienes levels which finally augments smooth muscle contraction [23]. Accordingly, 5-LOX is considered to be one of major casuses of bronchial asthma as well as other inflammatory diseases as allergic rhinitis and rheumatoid arthritis [24]. Analyzing of 5-LOX enzyme showed that it consists of seven anchors one (EH1), one hydrogen (H1), and fve van der Waal (V1 − V5). Both EH1 and H1 anchors are capable of make hydrogen bond interactions with the polar moieties. The substrate arachidonic acid does not involve in hydrogen bond interactions with the H1 anchor residues, which suggests that inhibitors with hydrogen-bonding interactions could have favorable binding affinity towards 5-LOX. The active site of 5-LOX is long and highly hydrophobic to accommodate the substrate arachidonic acid. The five van der Waals anchors in the catalytic site of 5-LOX, favors inhibitors with large aromatic moieties and long alkyl chains [25]. Accordingly, the presence of hydrophobic moieties and hydrogen bond acceptor/ donor moiety is preferable in the design of 5-LOX inhibitors.

Today, there has been a paradigm shift in the strategies of the anti-inflammatory drug development from single drug target to the magic shotgun “multiple drug target”. Consequently, the application of multitarget-directed ligands (MTDLs) in discovery of dual COX/5-LOX inhibitors is a hopeful approach to control inflammation in conditions that are aggrevated by the inhibition of only one target, e.g. asthma [26, 27].

**Fig. 2:**
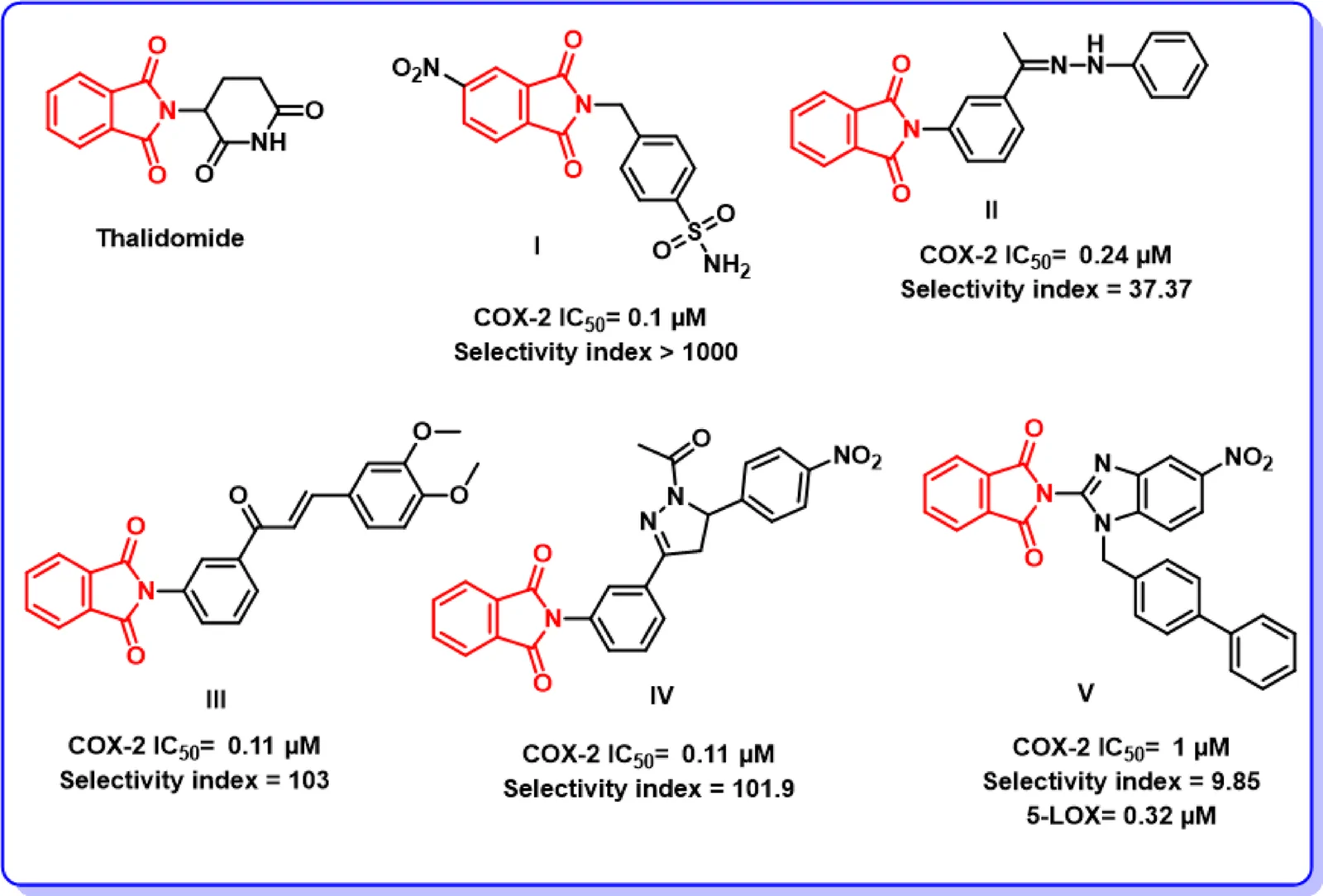
Phthalimide derivatives with COX-2/5-LOX inhibition activity

Phthalimides have received great attention due to their diverse biological activities such as anti-hyperlipidemic, anti-cancer and anti-inflammatory activities [28–30]. It is worth to mention that the bulky bicyclic ring system of phthalimide moiety may enhance COX-2 selectivity as it favours the entrance into the wide COX-2 active site rather than small one of COX-1 [31]. Thalidomide is a phthalimide derivative that inhibits both COX-1 and COX-2 isoenzymes [32] and also possesses an inhibitory effect on TNF-α production [33]. It is clearly defined that the phthalimide moiety in thalidomide is responsible for its biological activities while the toxicophore glutarimide subunit is the reason of the teratogenic effects [34]. Abdel-Aziz group has used fragment replacement to design and synthesize selective COX-2 phthalimide derivatives as compound **I** (Fig. 2) that maintains the pharmcophore features of celecoxib with extremely high selectivity index [30, 35, 36]. However many phthalimides that do not possess sulfonamide moiety displayed potent COX-2 inhibitory profile with good selectivity indicies. The phthalimide core hybrid with either hydrazono bridge **II**, chalcone moiety **III** or pyrazole ring **IV** have potent COX-2 inhibition activity but with lower selectivity index than celecoxib which is considred to be an advantage as explanied above [31]. In addition the phthalimide benzimidazole hybrid **V** inhibited both COX isoenzymes with moderate selectivity and also it was a potent inhibitor 5-LOX [34].

**Fig. 3:**
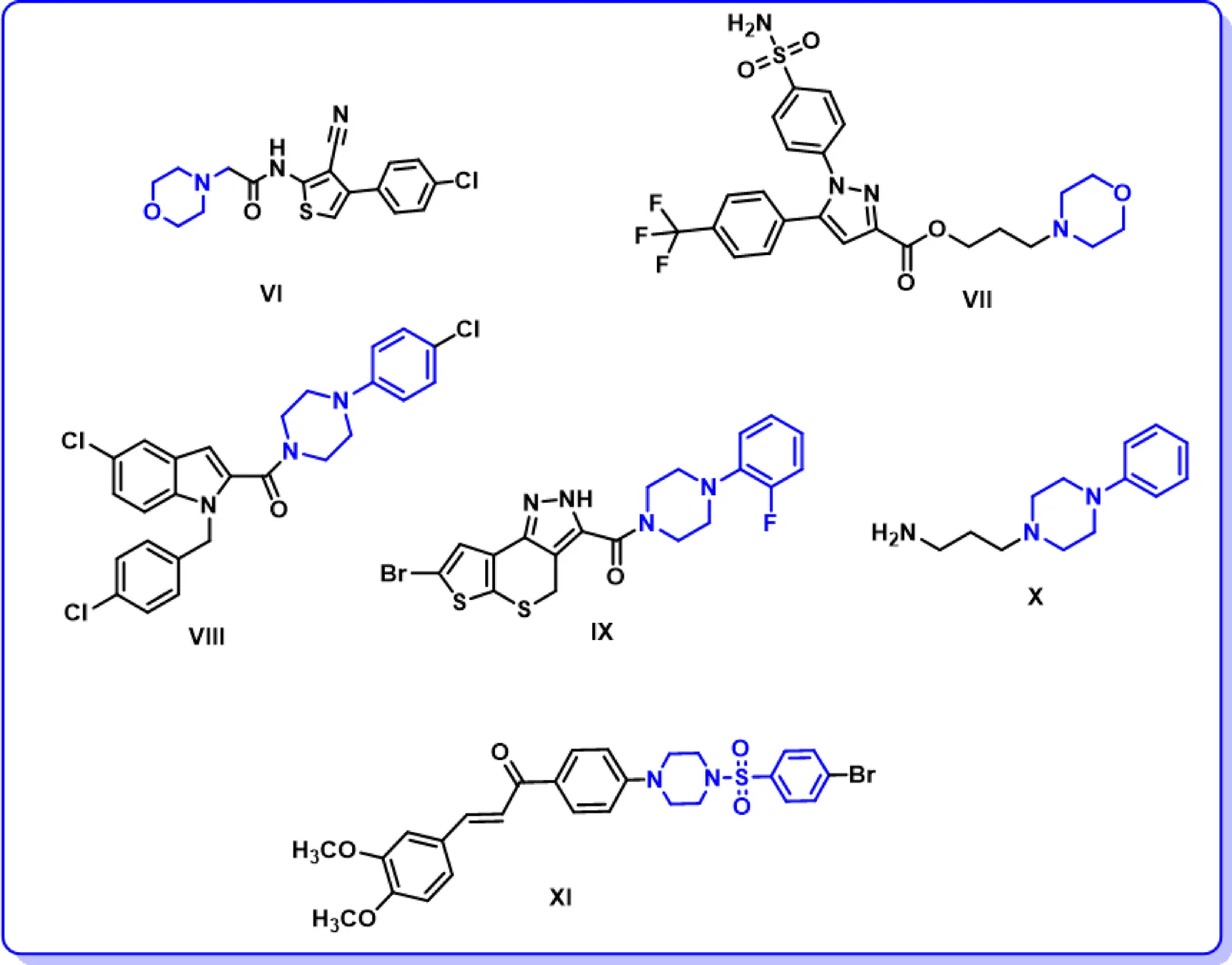
Morpholine and piperazine derivatives have COX-2/5-LOX inhibition activity

On the other hand, the pharmacological activities of the morpholine-carrying derivatives are of large significance in different biological fields. Many of them have dual COX-2/5-LOX inhibition effect such as morpholinoacetamide **VI** (Fig. 3) which has moderate selectivity index = 8.37 on COX-2 isoenzyme and also inhibits 5-LOX with IC_50_ value less than 5 µM [37]. Also, compound **VII** acts as COX-2/5-LOX with IC_50_ values = 28.52 µM, 0.21 µM and 0.75 µM on COX-1, COX-2, 5-LOX, respectively [38]. Moreover, the piperazine moiety acts as bioisotere for morpholine and also many piperazine derivatives have been found to exhibit a good anti-inflammatory activity. Huang et al. [39]. synthesized indole phenyl piperazine hybrid **VIII** that has a potent dual COX-2/5-LOX inhibitory effect with IC_50_ values of COX-2 = 0.03 µM and 5-LOX = 0.2 µM and moderate selectivity index (SI) (COX-1/COX-2) = 11.33. Also, the phenyl piperazine derivative **IX** act as 5-LOX inhibitor with IC_50_ = 5.7 µM compared to 5.3 µM of the reference drug [40]. Recently, two series of cyano and amino aryl piperazines were synthesized as dual COX-2/5-LOX inhibitors, among them 3-(4-phenylpiperazin-1-yl)propan-1-amine (**X**) displayed potent inhibition with IC_50_ = 8.73 µM and 2.45 µM on COX-2 and 5-LOX, respectively [41]. Additionally, the *N*-arylsulphonyl piperazines as compound **XI** displayed anti-inflammatory activity and significantly reduced in a dose-dependent manner the release of IL-6 and TNF-α in RAW264.7 macrophages treated with lipopolysaccharide (LPS) [42].

## Rational design of target compounds

**Fig. 4:**
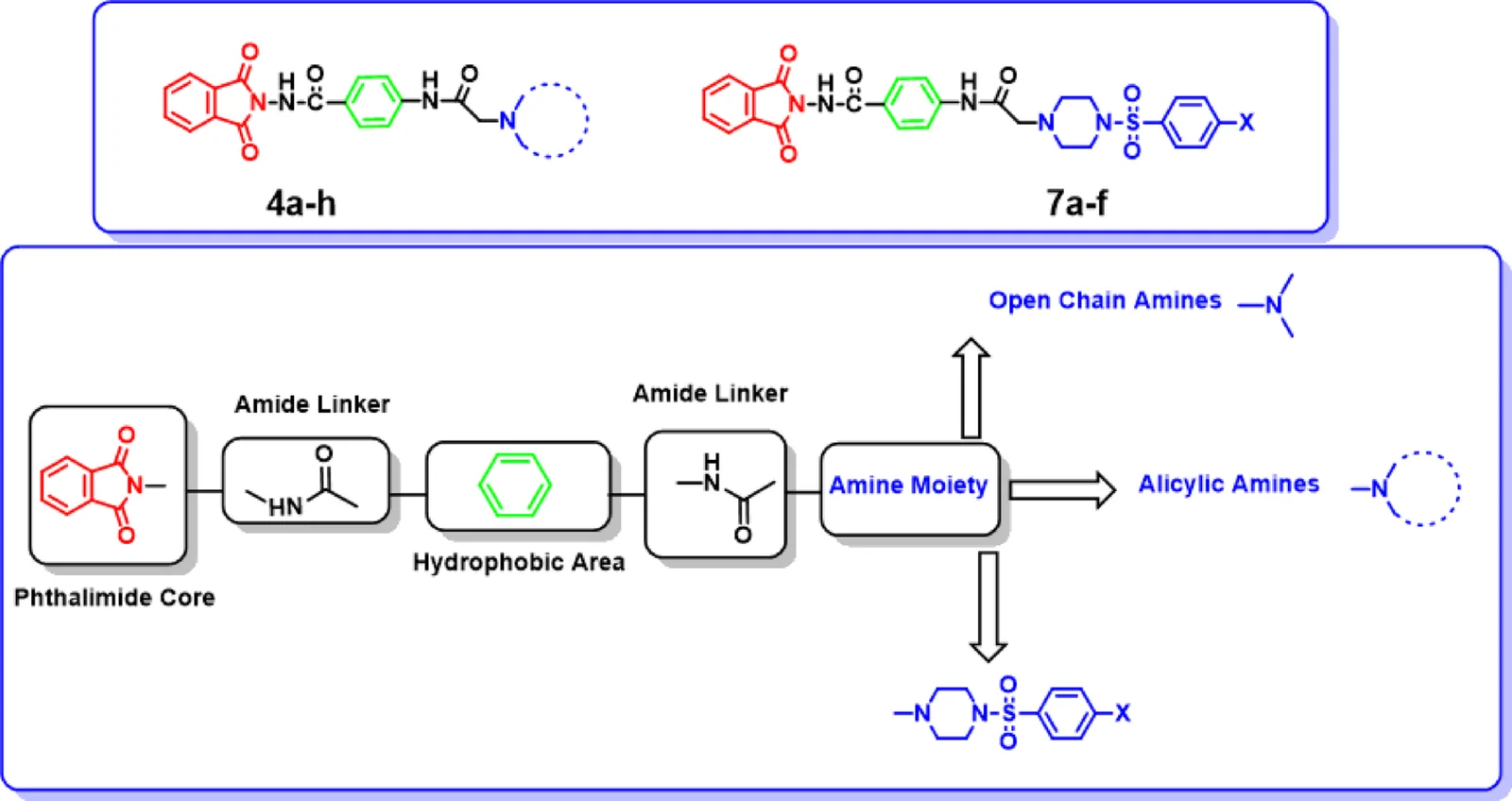
Design of the target compounds **4a-h** and **7a-f** as dual COX-2/5-LOX inhibitors

Based on the abovementioned premises and as a continuation of our previous studies to obtain new anti-inflammatory agents [43–46], two new series of phthalimides **4a-h** and **7a-f** (Fig. 4) were designed and synthesized to achieve dual COX-2/5-LOX inhibitors with moderately selective COX-2 inhibition to overcome the side effects of both highly selective COX-2 and non-selective COX inhibitors. The design mimics the previously reported pharmacophore models for the dual COX-2/5-LOX inhibitors [47, 48]. The design of the target compounds consists of the following pharmacophores: phthalimide core, central phenyl ring and different amine moieties that are connected together *via* two amide linkers. The phthalimide core and the central phenyl ring are expected to interact with hydrophobic binding region in COX-2 and the five van der Waal anchors of the active site of 5-LOX mimicking the interactions of the substrate arachidonic acid. In addition, the bulky bicyclic ring system of phthalimide may enhance COX-2 selectivity due to the perferential entrance into the wide COX-2 active site rather than small one of COX-1. Meanwhile, the two carbonyl groups of phthalimide ring act as hydrogen bond acceptor and could make hydrogen bonds with the amino acids located at the COX-2 specific hydrophilic side pocket mimicking the sulfonamide group of celecoxib. In addition these carbonyl groups could make hydrogen bonds with the residues of EH1 and H1 anchors in 5-LOX active site. Also, the two amide linkers also could form hydrogen bond interactions with the active site of both enzymes.

In series one **4a-h** the amine moiety was chosen from alicylic amine derivatives as morpholine that has dual COX-2/5-LOX inhibition activity and its bioisostere piperidine as well as *N*-phenyl piperazine, also the open chain aliphatic amine derivatives were synthesized to make a complete structure activity relationship study. In view of the structures of coxibs, where compounds having arylsulfonyl group show selective COX-2 inhibition activity, series two **7a-f** was synthesized with a sulphonyl group insertion based on the structure of *N*-phenyl piperazine derivative **4 h** from series one. The two series of the phthalimide derivatives **4a-h** and **7a-f** were tested in vitro to determine their inhibition effect of both COX-1 and COX-2 isoenzymes as well as 5-LOX. Also, the effect of the most active compounds on the production of the inflammatory mediators in RAW264.7 macrophages was determined.

## Results and discussion

### Chemistry

**Scheme 1:**
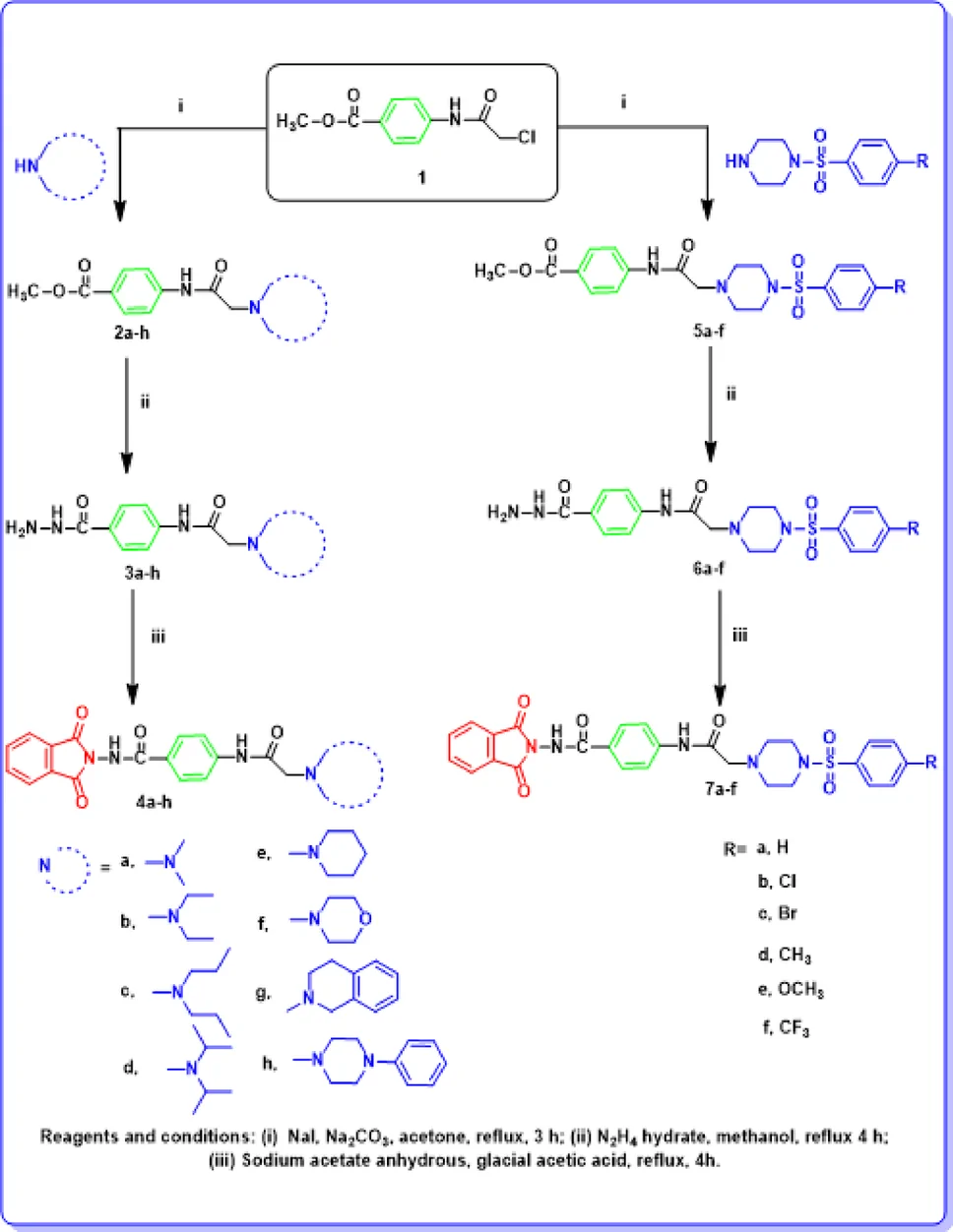
Synthesis of the target compounds **4a-h** and **7a-f**

The synthetic strategy of the target phthalimide derivatives is illustrated in Scheme 1. The starting material methyl 4-(2-chloroacetamido)benzoate (**1**) was obtained by the amidation of methyl 4-aminobenzoate using 2-chloroacetylchloride following the classical triethylamine catalyzed amidation [49]. The 1-((4-substituted phenyl)sulfonyl)piperazine derivatives were obtained by sulfonylation of piperazine with different benzenesulfonyl chloride derivatives in dichloromethane [50, 51]. The key intermediates **2a**-**h** and **5a**-**f** were prepared by the reaction of amide **1** with sodium iodide followed by nucleophilic replacement with the appropriate secondary amine or sulphonyl piperazine derivative. The ^1^H-NMR of the intermediates **2a**-**d** derived from aliphatic amines showed new upfield signals at range of *δ* 0.93-3 ppm. Meanwhile, the intermediates **5a**-**f** bearing phenyl sulphonyl piperazine moiety have signals at about *δ* 2.7 and 3.1 ppm in addition to the increment of the number of the downfield aromatic protons. Hydrazinolysis of the methyl ester intermediates **2a**-**h** and **5a**-**f** gave the penultimate compounds **3a**-**h** and **6a**-**f**, respectively. The chemical structures of the latter compounds were confirmed in ^1^H-NMR and ^13^C-NMR by disappearance of the signal of the methyl ester at about *δ* 3.8 and 51 ppm, respectively. In addition, the appearance of two new signals in ^1^H-NMR at about *δ* 4.4 and 9.8 ppm corresponding to NH_2_ and NH, respectively proved the formation of the acid hydrazide derivatives **3a**-**h** and **6a**-**f**. The target phthalimide derivatives **4a**-**h** and **7a**-**f** were prepared by condensation of the latter acid hydrazides with phthalic anhydride in glacial acetic acid and their structures were confirmed in ^1^H-NMR by disappearance of NH_2_ signal at about *δ* 4.4 ppm and also by increase in the number of aromatic protons corresponding to the 1,3-dioxoisoindoline ring. Also, a new signal appeared at about *δ* 165 ppm in ^13^C-NMR referring to the carbonyl of 1,3-dioxoisoindoline.

## Biological evaluation

In vitro **COX inhibition activity**

The capability of the two series of the newly synthesized phthalimide derivatives **4a**-**h** and **7a**-**f** to inhibit both COX-1 and COX-2 was determined using colorimetric enzyme immunoassay (EIA) kits. Celecoxib and meloxicam were used as selective COX-2 inhibitor reference drugs and indomethacin was used as non-selective COX inhibitor. The half-maximal inhibitory concentration (IC_50_, µM) values were determined and SI values were calculated as [IC_50_(COX-1)/IC_50_(COX-2)] to determine the safety profile of the compounds.

The results in Table 1 showed that all the tested compounds exhibited lower inhibitory activity toward COX-1 compared to indomethacin, suggesting better safety profile on GIT. It was found that compounds **4a**, **4c**, **4f**, **7a**, **7b** and **7f** displayed better inhibitory activity against COX-2 [IC_50_ = 0.99–6.28 µM] compared to the reference drug meloxicam [IC_50_ = 8.34 µM]. In addition, compound **4c** has IC_50_ = 0.99 µM which is comparable to that of celecoxib (IC_50_ = 0.82 µM). Fortunately, compounds **4c** and **7a** showed good inhibitory activity of COX-2 isoenzyme with IC_50_ values less than 10 µM with moderate SI which are higher than that of meloxicam and lower than that of celecoxib. These results are consistent with our design of the compounds to look for novel NSAIDs that are selective but not specific to COX-2 to reduce the cardiovascular risks associated with the use of highly selective COX-2 inhibitors.

## Structure activity relationship on COX-2

**Fig. 5:**
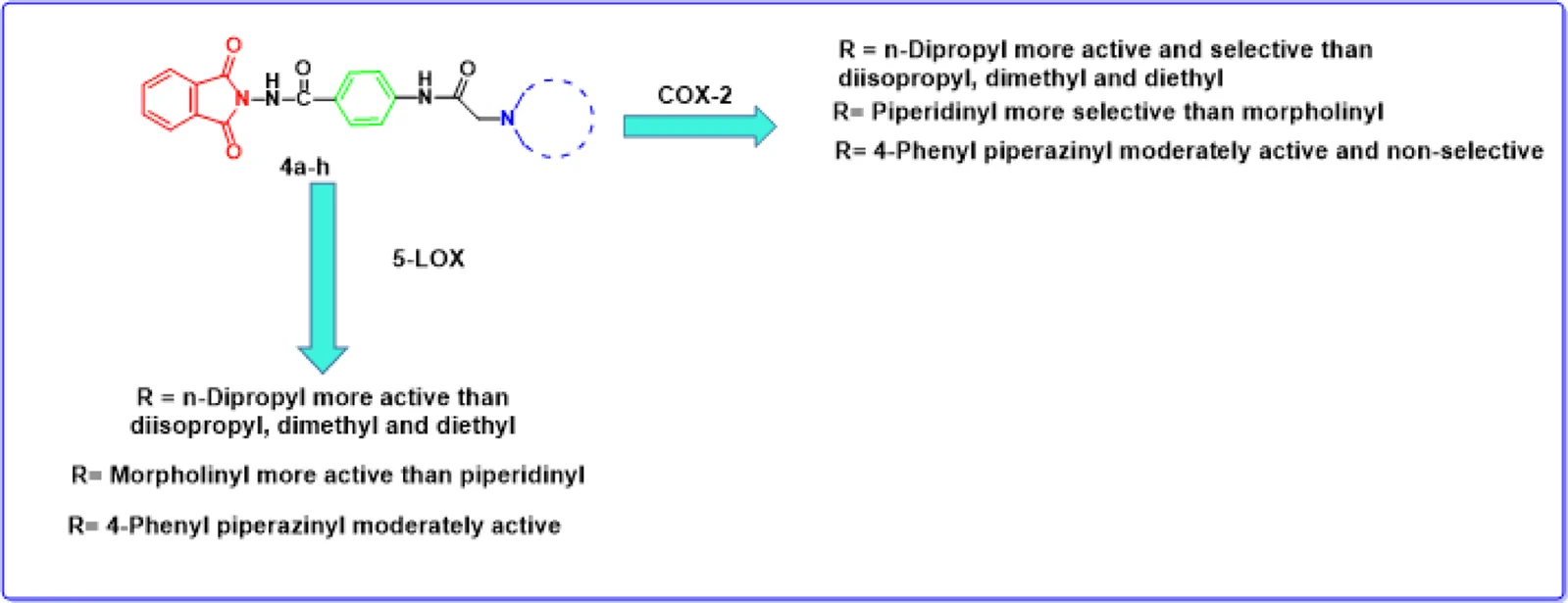
Structure activity relationship diagram illustrating the effect of the substitution pattern on **4a-h** as dual COX-2/5-LOX inhibitors

**Fig. 6:**
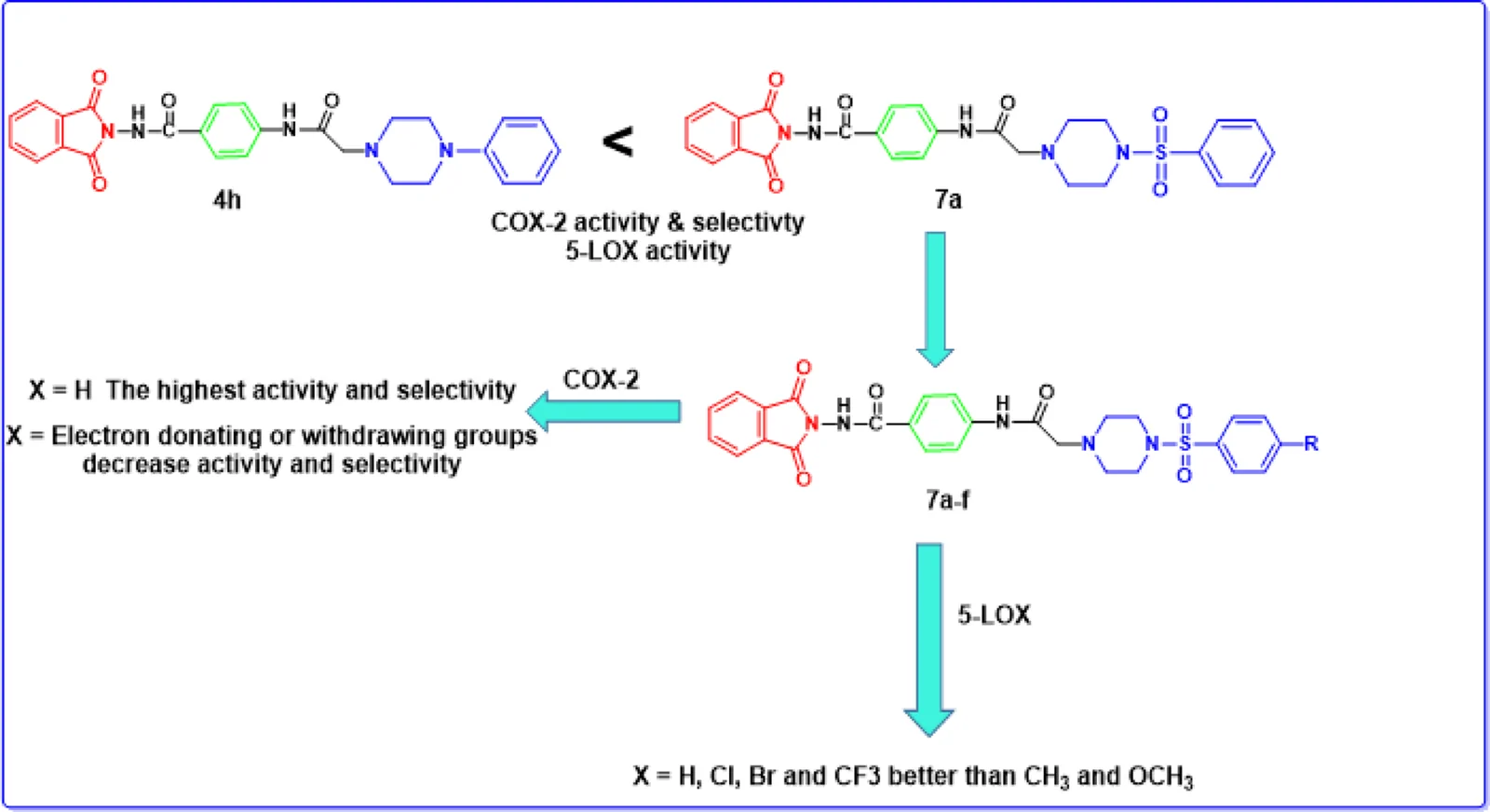
Structure activity relationship diagram illustrating the effect of the substitution pattern on **7a-f** as dual COX-2/5-LOX inhibitors

The outcomes of the structure activity relationship study are shown in Figs. 5 and 6. Analysis of the results in Table 1 revealed that compound **4c** which has a *n*-dipropyl amine moiety is the most active compound in both series in inhibition of COX-2 isoenzyme with moderate selectivity index 7.67. This compound is more potent than the reference drug meloxicam on COX-2 by about 8-fold. Meanwhile, the activity and selectivity on COX-2 of its branched isomer **4d** reduced by about 43 and 5.21-fold, respectively. Also, the activity of the dimethyl and diethyl analogues **4a** and **4b** was less than the *n*-dipropyl derivative **4c** by 3 and 11-fold, respectively. It was notable that dimethyl analogue **4a** could achieve good inhibition activity of COX-2 with IC_50_ = 3.1 µM but unfortunately with non-selective effect (SI = 0.57). Regarding compounds **4e-h** that have alicylic amine side chain, it was observed that both piperidine and 1,2,3,4-tetrahydroisoquinoline derivatives **4e** and **4 g** have moderate COX-2 inhibition effect with IC_50_ values equal or less than 15.4 µM with selectivity indices less than celecoxib and higher than that of meloxicam. Meanwhile, the replacement of piperidine ring in compound **4e** with its morpholine isostere in compound **4f** led to an increase of activity on COX-2 by about 3-fold, but this was accompanied by reduction of selectivity by 3.6-fold. It was remarkable that the phenyl piperazine derivative **4 h** was less active by 2-fold compared to the fused analogue **4 g**.

Regarding the second series **7a-f**, it was found that the insertion of sulphonyl group between piperazine ring and phenyl moiety of compound **4 h** resulted in a dramatically increase of both activity and selectivity on COX-2 by 18 and 15-fold, respectively as observed in compound **7a**. The latter compound is more potent on COX-2 isoenzyme than the reference drug meloxicam by 4.8-fold and its selectivity is higher than meloxicam but lower than celecoxib. The *p*-chloro phenyl analogue **7b** has a comparable activity on COX-2 as its unsubstituted derivative **7a** but with a reduction of selectivity by 3.7-fold. Also, compound **7f** that has an electron withdrawing group (CF_3_) displayed good activity on COX-2 with IC_50_ less than 10 µM with selectivity slightly higher than meloxicam. Both the *p*-bromo and *p*- methoxy phenyl derivatives **7c** and **7e** have comparable moderate activity on COX-2 with IC_50_ less than 30 µM. However, the *p*-bromo derivative **7c** is slightly more selective than *p*-methoxy derivative **7e**. These results indicated that substitution on phenyl group in compounds **7a**-**f** with either electron donating or withdrawing groups reduced both the activity and selectivity of compound **7a**.

In vitro **5-LOX inhibition activity**

The compounds of the two series **4a**-**h** and **7a**-**f** were also tested for 5-lipoxygenase inhibition activity and zileuton was used as reference standard. Compounds **4c**, **4f**, **7a** and **7f** exhibited potent inhibitory activity with IC_50_ values less than 1 µM. Compound **4c** is more active than the reference drug zileuton by 1.3 fold, while compound **4f** is equipotent to zileuton. Meanwhile, the compounds of the second series **7a** and **7f** are less potent than zileuton. Compounds **4a**, **4b** and **7b** had moderate activity with IC_50_ values below 10 µM. The results showed that compounds **4a**, **4b** and **7b** are less active than the reference drug zileuton by 4, 13, 2.4 fold, respectively. In addition compounds **4d**, **4 g**, **4 h** and **7c** displayed a mild inhibition activity on 5-LOX enzyme with IC_50_ values less than 20 µM and they are less active than zileuton. The rest of the compounds **4e**, **7c**, **7d** and **7e** have poor inhibitory activity on 5-LOX.

## Structure activity relationship on 5-LOX

The results in Table 1 showed that compound **4c** which has a *n*-dipropyl amine moiety is the most active compound in both series in inhibition of 5-LOX. However, the activity dropped by about 27 fold in its branched isomer **4d**. Also, the decrease of the length of the alkyl side chain reduced the activity by 5 and 7-fold in compounds **4a** and **4b**, respectively. Concerning the derivatives of the alicylic amines **4e-h**, it was found the morpholine derivative **4f** is the most active one with IC_50_ value equal to zileuton. Meanwhile, the activity of compound **4f** declined by 50-fold in its piperidine isostere **4e**. Fortunately, the activity of **4e** enhanced by about 3-fold upon introducing an aromatic moiety to the piperidine ring in compound **4 g**. The phenyl piperazine **4 h** was less active by 1.5-fold in comparison with the fused analogue **4 g**.

In the second series **7a-f**, it was notable that the positioning of sulphonyl group between piperazine ring and phenyl moiety of compound **4 h** improved the inhibition effect on 5- LOX by about 16-fold in compound **7a**. Compound **7f** that possesses an electron withdrawing group (CF_3_) is slightly more potent than its unsubstituted congener **7a**. Also, the *p*-chloro phenyl derivative **7b** has a comparable inhibition activity as its unsubstituted analogue **7a**. The derivatives **7d** and **7e** that have electron donating groups (OCH_3_ and CH_3_) are less active than their unsubstituted analogue **7a** by 64 and 26-fold, respectively.

**Table 1:**
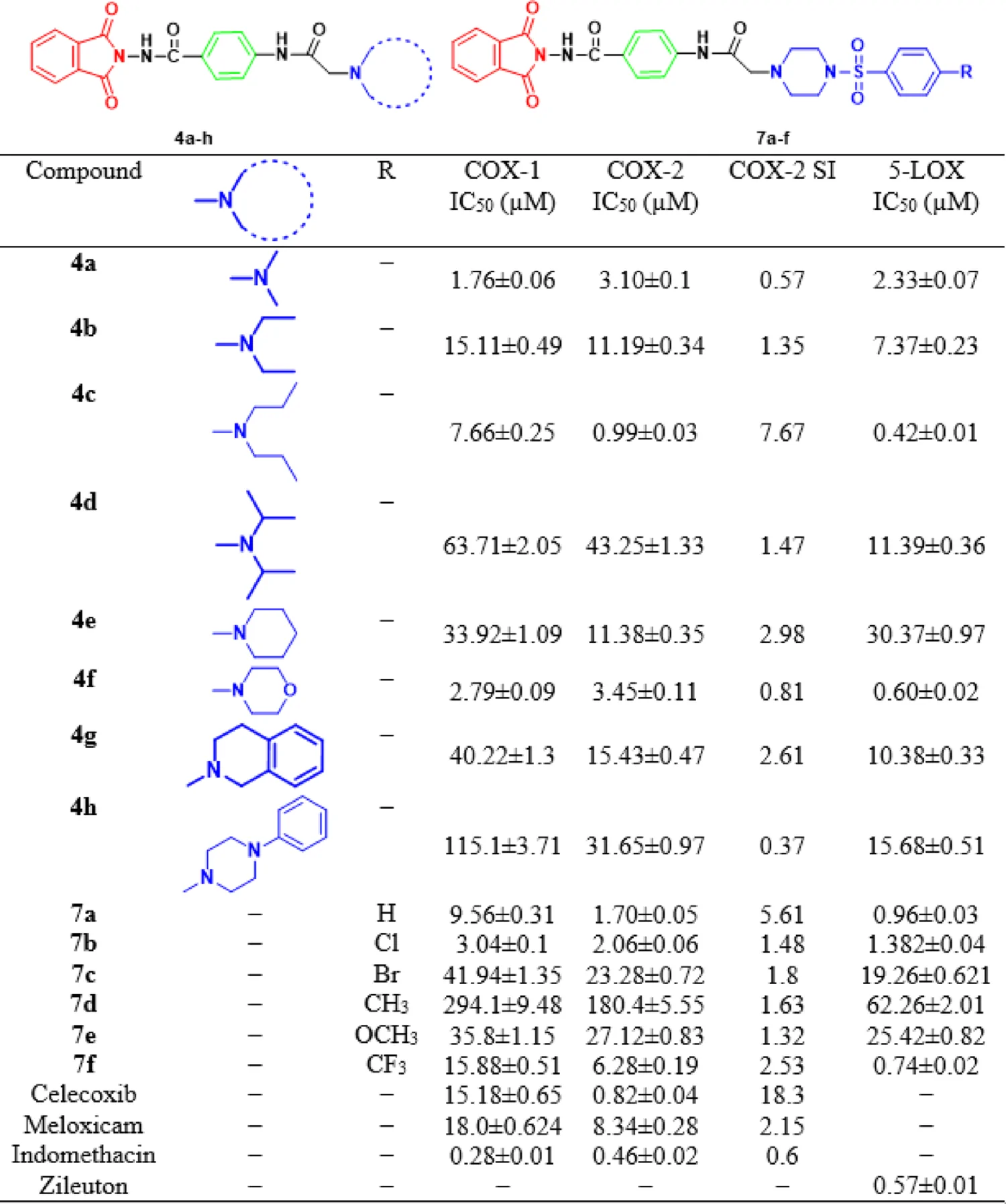
COX and 5-LOX inhibitory activities of the target compounds **4a**-**h** and **7a**-**f**

Data expressed as mean ± SEM (*n* = 3). COX-2 SI is calculated as (IC_50_ COX-1/COX-2)

### Inhibition of NO production in RAW 264.7 macrophages

Nitric oxide (NO) has a key role in the progress of inflammation although it acts as an anti-inflammatory agent under normal physiological conditions [52]. The excess release of NO during inflammation can be harmful. NO reacts with superoxide to produce peroxynitrite that has a highly reactive oxidant effects that encourages lipid peroxidation, DNA damage and protein nitration which lead finally to tissue damage [53]. This may exacerbate inflammatory damage, disrupt regular cellular processes and potentially trigger necrosis or apoptosis. Also, NO dysregulation is a major disadvantage in inflammatory processes because high levels of NO are linked to chronic inflammatory disorders such as rheumatoid arthritis, sepsis as well as neurodegeneration [54].

All the compounds of the two series **4a-h** and **7a-f** were evaluated for their inhibitory activity towards NO production in LPS-induced RAW 264.7 macrophages. Before the determination of the ability of the target compounds to suppress the induced RAW 264.7 macrophage’s production of NO, the effect of the compounds on macrophage’s viability was first assessed. This was performed to guarantee that the achieved results are due to specific rather than non-specific inhibition since the latter might result from macrophages death. It was found that all of the tested compounds have no cytotoxic effect on RAW 264.7 macrophages and have IC_50_ value more than 200 µM. The effect of the target compounds on NO production inhibition at of 100 µM and IC_50_ values for the most actives are expressed in Table 2. All of the compounds under investigation except compound **4b** could decrease the NO production by more than 40% at 100 µM. It was observed in the first series **4a**-**f** that the piperidinyl derivative **4e** and its fused aromatic analogue **4 g** are the most active derivatives with IC_50_ values equals 82.5 µM and 64.9 µM, respectively. It was found that the introducing of sulphonyl group between piperazine ring and phenyl moiety of compound **4 h** improved the inhibitory effect on NO production in compound **7a**. It was notable that all the compounds of the second series **7a-f** except the *p*-chlorophenyl derivative **7b** have similar inhibitory activity with IC_50_ values equal or less than 75 µM.

**Table 2:** Effect of the target compounds **4a-h** and **7a-f** on NO production and cell viability

Compound	% Inhibition (100 µM)	IC_50_ (µM)	Cell viability IC_50_ (µM)
**4a**	47.52 ± 1.22	> 100	> 200
**4b**	35.81 ± 1.15	> 100	> 200
**4c**	45.44 ± 1.41	> 100	> 200
**4d**	42.92 ± 0.95	> 100	> 200
**4e**	50.14 ± 2.01	82.52 ± 5.63	> 200
**4f**	41.93 ± 1.19	> 100	> 200
**4 g**	61.92 ± 1.55	64.91 ± 3.33	> 200
**4 h**	49.72 ± 2.04	> 100	> 200
**7a**	64.25 ± 3.12	68.09 ± 3.21	> 200
**7b**	58.19 ± 1.29	> 100	> 200
**7c**	62.41 ± 1.13	75.62 ± 5.12	> 200
**7d**	63.72 ± 1.75	60.1 ± 4.99	> 200
**7e**	57.51 ± 2.11	74.6 ± 4.54	> 200
**7f**	63.97 ± 2.40	67.6 ± 3.99	> 200

Data expressed as mean ± SD (*n* = 3)

## Effects on inflammatory gene expression in LPS- stimulated RAW 264.7 macrophages

Compounds **12c** and **13a** that have both COX-2 selective inhibition effect as well as LOX-5 suppression activity were selected to determine their effects on inflammatory gene expression in LPS-stimulated RAW 264.7 macrophages by RT-PCR at 10 µM (Table 3). PGE-2 is an important pro-inflammatory mediator resulted from AA metabolism *via* COX enzymes. Compounds **4c** and **7a** caused a reduction of PGE-2 mRNA expression by 0.432-fold and 0.698-fold, respectively, compared to the control LPS-stimulated RAW 264.7 macrophages. IL-6 is a pro-inflammatory cytokine that has an important effect in the controlling of inflammation as well as immune response. In the LPS-stimulated macrophages the expression of IL-6 is increased. In spite of the IL-6 is not a direct product of AA metabolism by 5-LOX, but its expression is enhanced by leukotrienes which are produced by the 5-LOX pathway [55]. Treatment with compounds **4c** and **7a**, resulted in downregulation of IL-6 mRNA by 0.237-fold and 0.318-fold, respectively compared to the control LPS-stimulated RAW 264.7 macrophages. Furthermore, TNF-ɑ is also a pro-inflammatory cytokine that is increasd in macrophages following LPS treatment. It has been reported that prostaglandins and leukotrienes enhance TNF-α production [56]. Also, compounds **4c** and **7a** reduced the level of TNF-α mRNA by 0.331-fold and 0.527-fold, respectively in comparison with the control LPS-treated cells. These results indicates that compound **4c** has more pronounced effect than compound **7a** on the reduction of the measured inflammatory mediators PGE-2, IL-6 and TNF-α supporting its potential anti-inflammatory effect in macrophages.

**Table 3:** Effect of compounds **4c** and **7a** at 10 µM on the expression of PGE-2, IL-6 and TNF-α in the LPS- stimulated RAW 264.7 macrophages

Compound	PGE-2^a^	IL-6^a^	TNF-α^a^
**4c**	0.432	0.237	0.331
**7a**	0.698	0.318	0.527
LPS	1	1	1

^a^ : Fold change

## Molecular docking

Molecular docking for compounds **4c** and **7a** which are the most active derivatives on both COX-2 (PDB ID: 5KIR) and 5-LOX (PDB ID: 6N2W) was carried out to determine their binding interactions.

### Molecular docking on COX-2

**Fig. 7:**
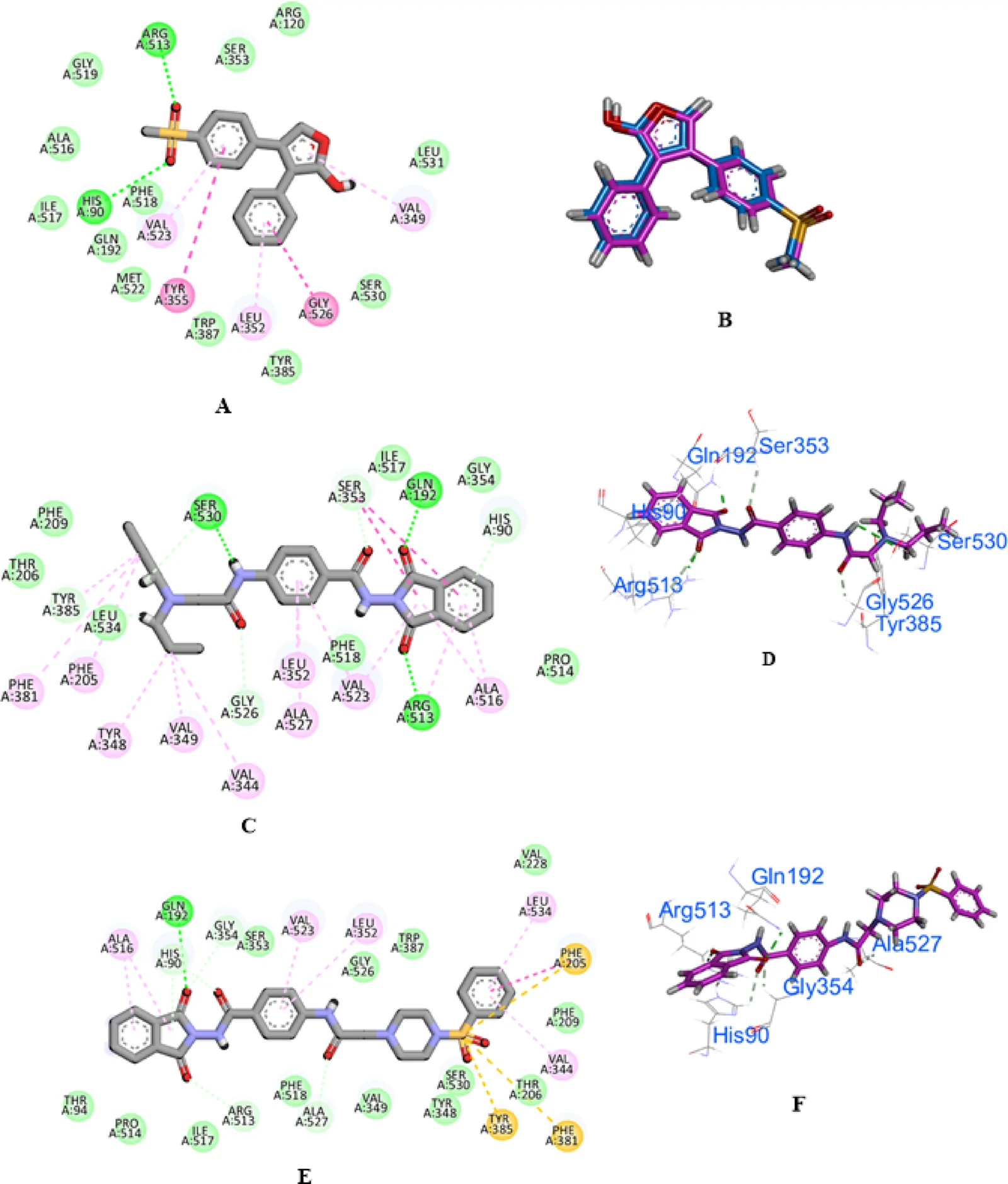
**A**. Interactions of the native ligand (rofecoxib) within the active site of COX-2. **B.** Superimposition of the co-crystalized and the re-docked pose of the native ligand in the active site of COX-2. **C**. 2D Binding mode of compound **4c** into the active site of COX-2. **D**. 3D Binding mode of compound **4c** into the active site of COX-2. **E**. 2D Binding mode of compound **7a** into the active site of COX-2. **F**. 3D Binding mode of compound **7a** into the active site of COX-2

The catalytic site of COX-2 isoenzyme has a hydrophobic channel that accommodates the fatty acid substrate as well as hydrophobic parts of the ligands. The active site of COX-2 is distinguished from COX-1 by increase in volume as mentioned above which led to creation of a hydrophilic side pocket off the main channel responsible of COX-2 selectivity with Arg513 located at its base [57]. In order to check the validity of the used docking protocol, re-docking of the co-crystallized ligand (rofecoxib) in the active site of COX-2 was carried out. The oxygen atoms of the methylsulfone moiety of the re-docked pose could attain the same hydrogen bond interactions with key amino acids His90 and Arg513 in the hydrophilic side pocket [58] with RMSD = 0.433 Å (Fig. 7A and B). The RMSD less than two indicated the confidence of the docking protocol to be applied for the test compound [59–62]. The re-docked pose of the co-crystallized ligand has C-Docker interaction energy =-41.78 kcal/mol.

The results of docking demonstrated that compound **4c** had C-Docker interaction energy =-40.91 kcal/mol. Docking of compound **4c** in the active site of COX-2 showed presence of two hydrogen bonds between the oxygen of the carbonyl groups of phthalimide moiety and Gln192 as well as Arg513 in the side pocket. Also, His90 made a non-conventional hydrogen bond. Moreover, Ser530 which is crucial in many other COX-2 inhibitors [15, 63] made conventional and non-conventional hydrogen bonds with NH and propyl side chain, respectively. In addition, Ser353, Tyr385 and Gly526 made three non-conventional hydrogen bonds. The compound is further stabilized in the active site through hydrophobic interactions between the phthalimide core, central phenyl ring and the dipropyl side chains with amino acids Phe205, Val344, Tyr348, Val349, Phe381, Tyr385, Leu352, Ser353, Arg513, Ala516, Val523 and Ala527 (Fig. 7C and D).

The carbonyl group of phthalimide moiety in compound **7a** formed one conventional hydrogen bond with Gln192 in the selectivity pocket. Also, Arg513 and His90 in the selectivity pocket made three non-conventional hydrogen bonds. In addition, the compound made hydrogen bond with amino acid Gly354 that was reported in the interactions of other COX-2 inhibitors [26]. It was found that the carbonyl of the amide linker acts as hydrogen bond acceptor and made one hydrogen bond Ala527. The sulfur of sulphonamide group made pi-sulfur interactions with the phenyl ring of the aromatic amino acids Phe205, Phe381 and Tyr385. The compound is also stabilized in the binding site *via* hydrophobic interactions with Phe205, Val344, Leu352, Ala516, Val523 and Leu534 (Fig. 7E and F). This compound has a C-Docker interaction energy =-40.71 kcal/mol which is comparable to that of compound **4c**.

### Molecular docking on 5-LOX

**Fig. 8:**
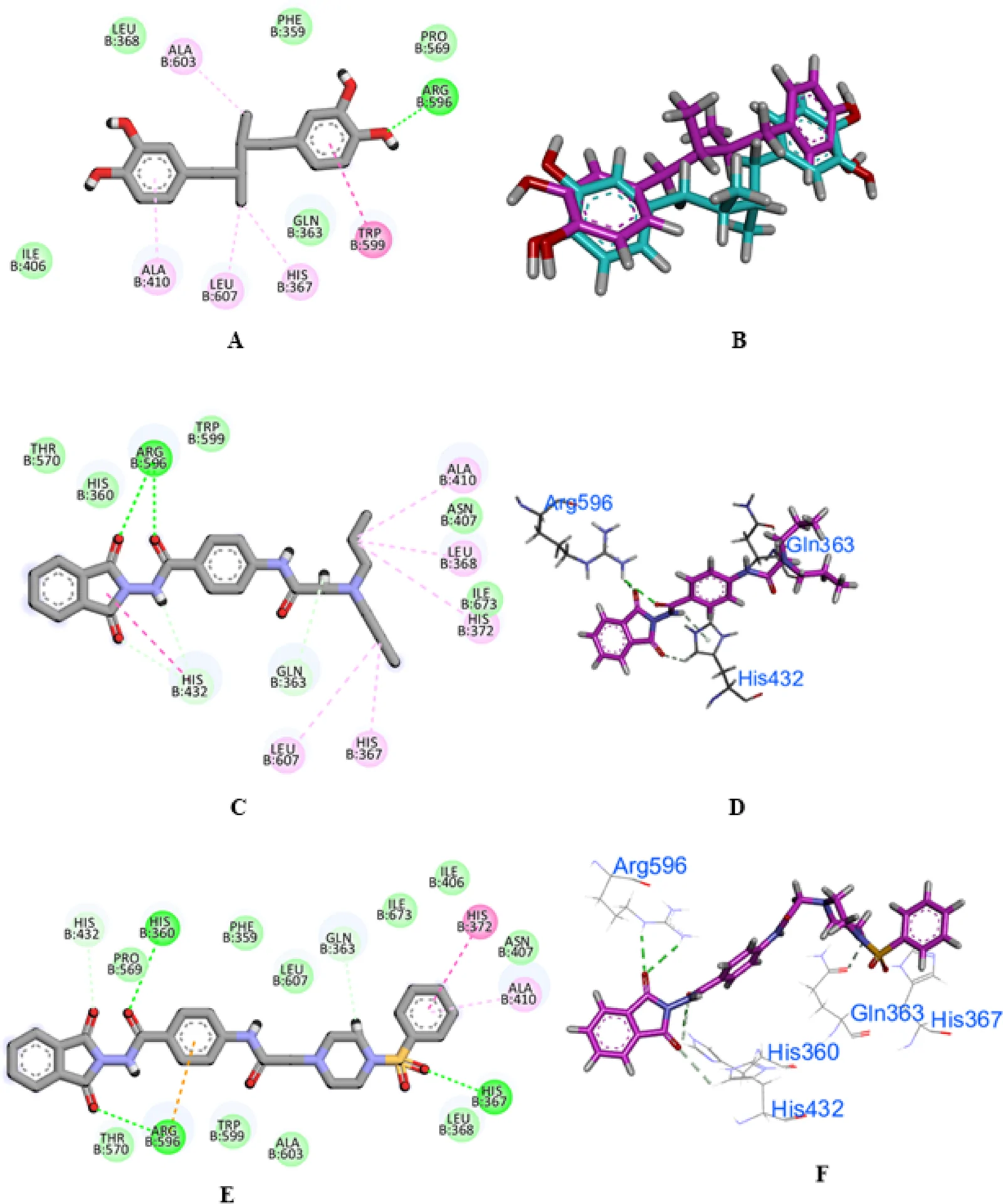
**A**. Interactions of the native ligand (nordihydroguaiaretic acid) within the active site of 5-LOX. **B.** Superimposition of the co-crystalized and the re-docked pose of the native ligand in the active site of 5-LOX. **C**. 2D Binding mode of compound **4c** into the active site of 5-LOX. **D**. 3D Binding mode of compound **4c** into the active site of 5-LOX. **E**. 2D Binding mode of compound **7a** into the active site of 5-LOX. **F**. 3D Binding mode of compound **7a** into the active site of 5-LOX

As mentioned above prior to docking of the test compound a validation step for the used docking protocol should be performed. It was found that the re-docked ligand performed the key hydrogen bond with Arg596 as the co-crystallized ligand (nordihydroguaiaretic acid) with RMSD = 1.526 Å (Fig. 8A and B) with C-Docker interaction energy =-43.78 kcal/mol. The results of docking demonstrated that compound **4c** had C-Docker interaction energy =-44.65 kcal/mol and the carbonyl groups of the amide as well as the phthalimide ring act as hydrogen bond acceptors and perform two hydrogen bonds with the essential amino acid Arg596. Furthermore, the compound was stabilized with three additional non-classical hydrogen bonds with His432 and Gln363 which are important for binding of other 5-LOX inhibitors [64, 65]. As previously mentioned, the catalytic site of 5-LOX has five van der Waals anchors which give indication that the catalytic site is hydrophobic in nature and may have a preference for inhibitors with large aromatic moieties or long aliphatic chains that mimic the substrate arachidonic acid [25]. It was found that the two dipropyl side chains of compound **4c** made hydrophobic interactions with residues His367, Leu368, His372, Ala410 and Leu607, also the phathalimide moiety interacted with His432 *via* hydrophobic interaction (Fig. 8C and D). Also, the carbonyl group of the phthalimide moiety in compound **7a** acts as hydrogen bond acceptor and formed hydrogen bond with the key amino acid Arg596. The latter amino acid is involved as well in electrostatic interaction with the central phenyl ring. Similar to compound **4c** it was found that His432 and Gln363 made non-classical hydrogen bonds. In addition, the carbonyl of the amide linker and SO_2_ group act as hydrogen bond acceptor and formed hydrogen bonds with His360 and His367, respectively. Furthermore, the distal phenyl ring has hydrophobic interactions with His372 and Ala410 (Fig. 8E and F). This compound has a C-Docker interaction energy =-44.92 kcal/mol.

### Molecular dynamic simulations

#### Molecular dynamic simulations on COX-2

**Fig. 9:**
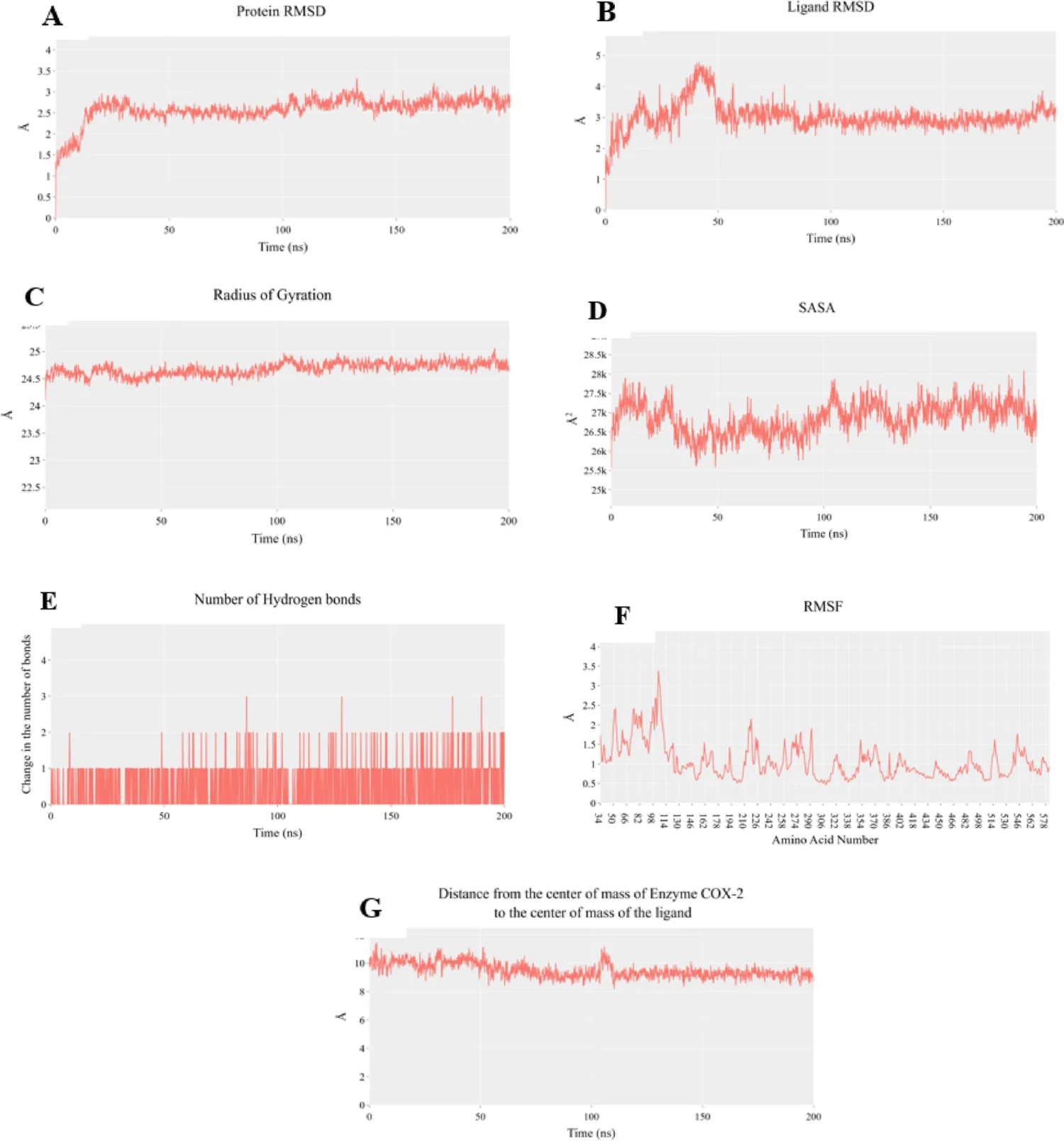
**A** RMSD values from the trajectory for the enzyme COX-2, **B** The ligand RMSD values, **C** radius of gyration for the Enzyme COX-2 protein, **D** SASA for enzyme COX-2, **E** Change in the number of hydrogen bonds between compound **4c** and enzyme COX-2, **F** RMSF for enzyme COX-2 protein, **G** Distance from the center of mass of compound **4c** and enzyme COX-2

Conformational stability of complex between the compound **4c** and COX-2 was evaluated over a 200 ns production trajectory. Backbone Cα RMSD increased during the initial 15 ns, plateauing at 2.5 Å for the first 100 ns and subsequently slightly rising to 2.7 Å (Fig. 9A), indicating rapid equilibration and sustained structural integrity. The ligand heavy-atom RMSD exhibited a pronounced increase within the first 45 ns, reaching a transient maximum of 4.6 Å before converging to 3.0 Å (Fig. 8B), implying limited conformational drift while remaining attached to the binding pocket. Global metrics verified these observations; the radius of gyration (Rg) remained essentially constant at an average of 24.7 Å (Fig. 9C), and the solvent-accessible surface area (SASA) oscillated narrowly around 26,750 Å^2^ (Fig. 9D), indicating a compact, folded state. Hydrogen-bond analysis revealed a dominant single persistent contact between **4c** and the protein, with some frames showing formation of two or three hydrogen bonds (Fig. 9E). Per-residue Cα RMSF values were ≤ 1.5 Å across most of the structure, with a localized maximum of 3.4 Å confined to residues Lys97–Asn110 in the N-terminal domain (Fig. 9F). The protein–ligand center-of-mass distance decreased monotonically over the first 50 ns and stabilized at 9.0 Å thereafter (Fig. 9G), confirming a stable binding mode after initial equilibration. Collectively, these metrics indicate that the COX-2_**4c** complex maintains global structural rigidity, with flexibility restricted to the N-terminal segment and the ligand remaining firmly bound to the binding site.

**Fig. 10:**
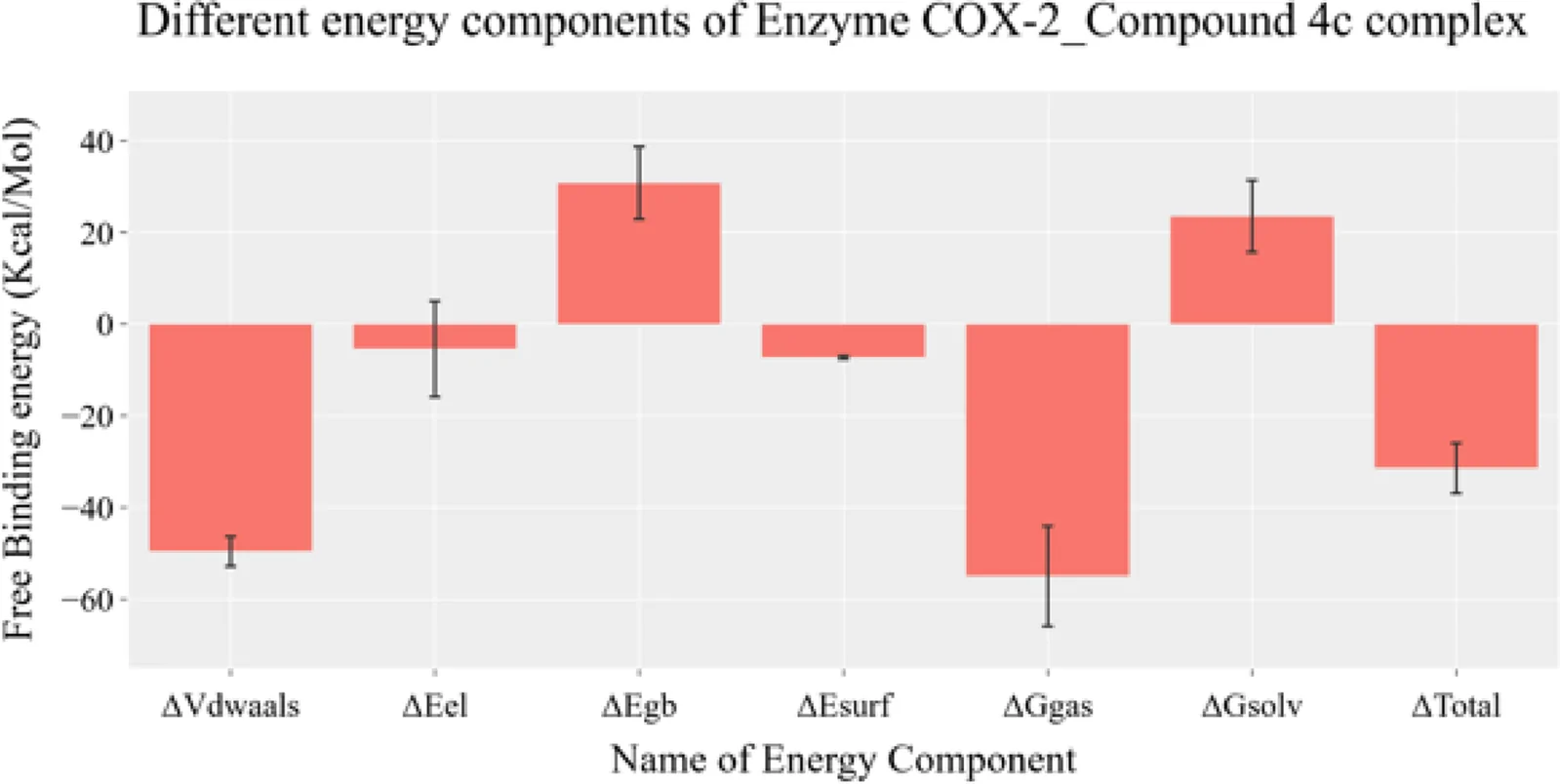
The different energetic components of MM-GBSA and their values for the system. Bars represent the standard deviations

**Fig. 11:**
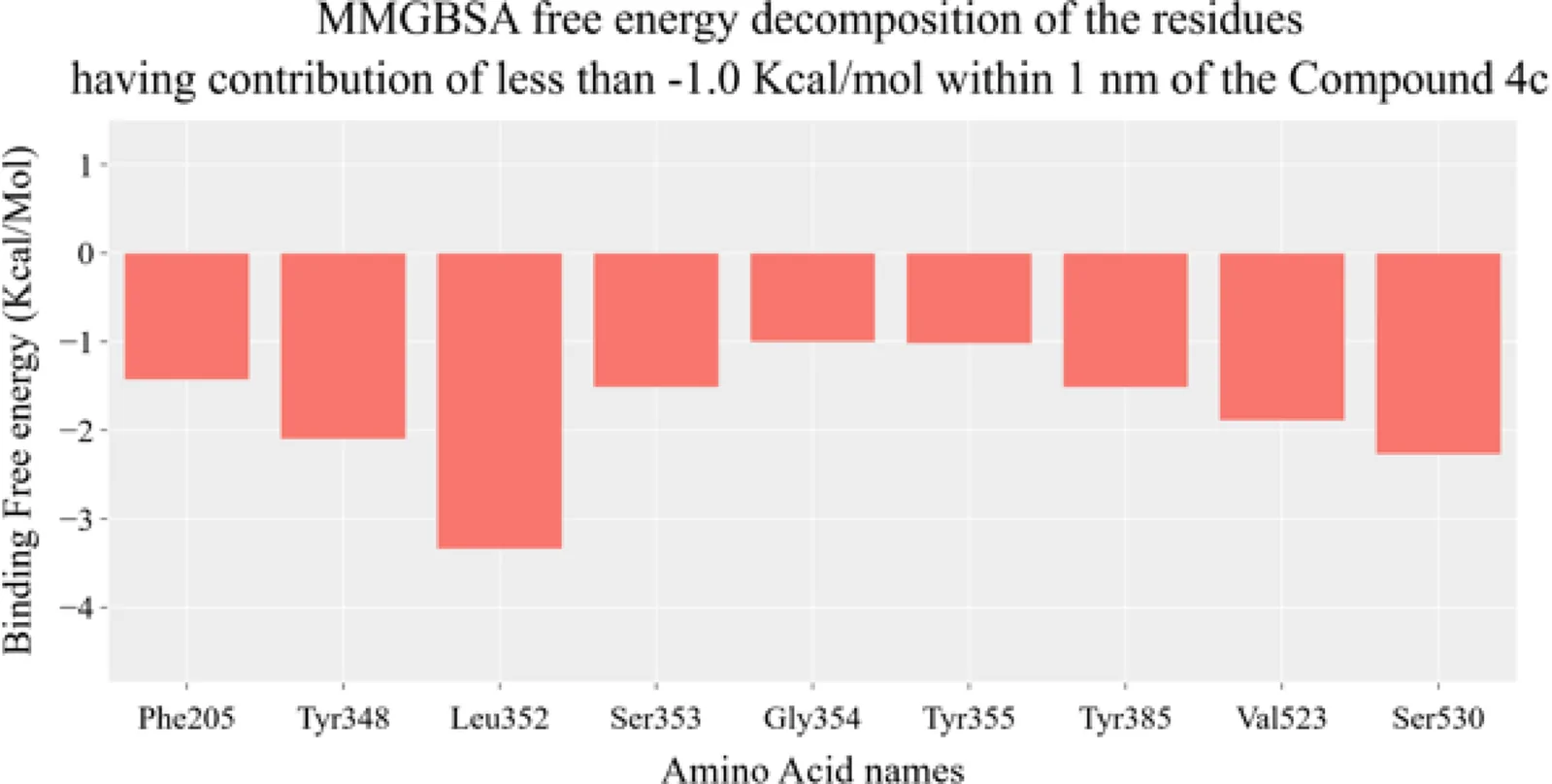
The binding free energy decomposition of the enzyme COX-2_compound **4c** complex. Amino acids shown are the ones within 1 nm of the ligand, and having a binding affinity of -1 Kcal/mol or better

MM-GBSA calculations yielded an average binding free energy ΔG of − 31.42 kcal mol⁻¹ (Fig. 10). Decomposition of the energetic components revealed that the interaction is dominated by van der Waals forces (–49.57 kcal mol⁻¹), whereas electrostatic interactions contributed modestly (–5.41 kcal mol⁻¹). The polar solvation term was strongly unfavourable (+ 30.82 kcal mol⁻¹), while the non-polar solvation showed a favourable contribution (–7.26 kcal mol⁻¹). Per-residue free-energy decomposition within a 1 nm sphere of the ligand identified nine residues whose individual contributions exceeded − 1 kcal mol⁻¹: Phe205 (–1.42 kcal mol⁻¹), Tyr348 (–2.09 kcal mol⁻¹), Leu352 (–3.33 kcal mol⁻¹), Ser353 (–1.51 kcal mol⁻¹), Gly354 (–1.00 kcal mol⁻¹), Tyr355 (–1.01 kcal mol⁻¹), Tyr385 (–1.51 kcal mol⁻¹), Val523 (–1.88 kcal mol⁻¹), and Ser530 (–2.27 kcal mol⁻¹) (Fig. 11).

**Fig. 12:**
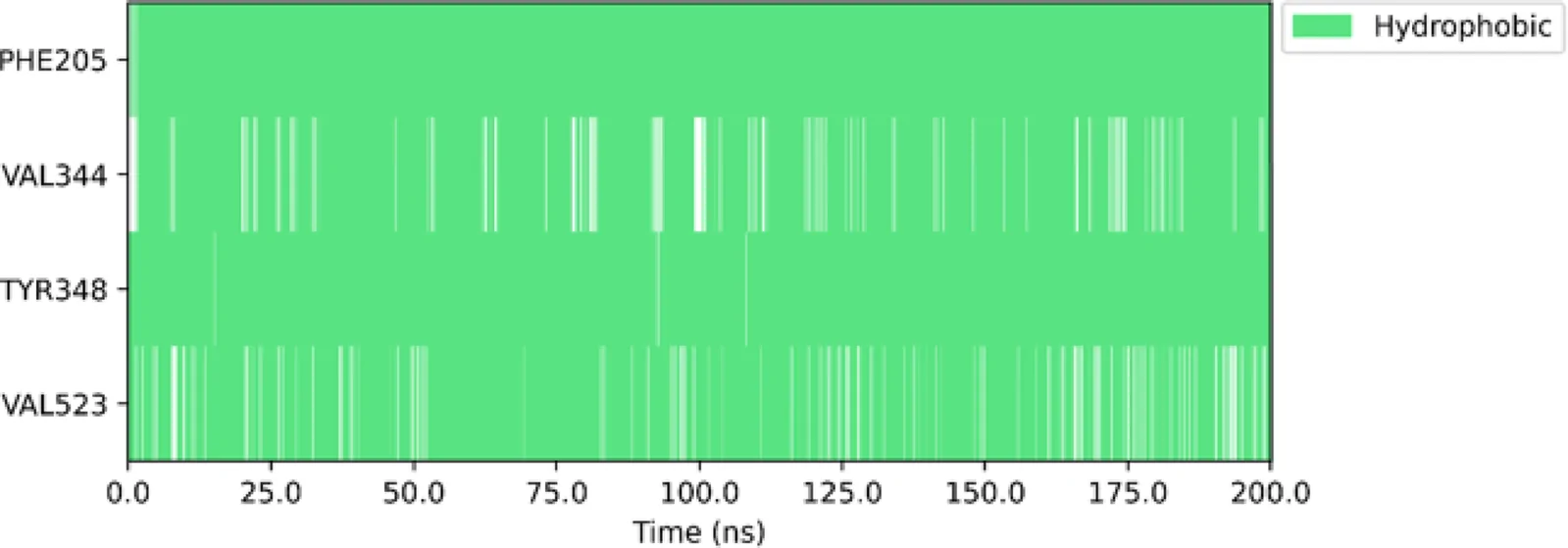
The amino acids, the types of interactions with the compound **4c**, and their occurrence during the whole simulation time using the ProLIF Python library

Persistent non-covalent interactions were further quantified with Protein-Ligand Interaction Fingerprints (ProLIF). Hydrophobic contacts show > 90% occupancy for Phe205 (99.7%), Val344 (90.85%), Tyr348 (99.85%), and Val523 (90.85%) (Fig. 12), underscoring the central role of these residues in stabilizing the ligand within the COX-2 active site through sustained hydrophobic complementarity.

**Fig. 13:**
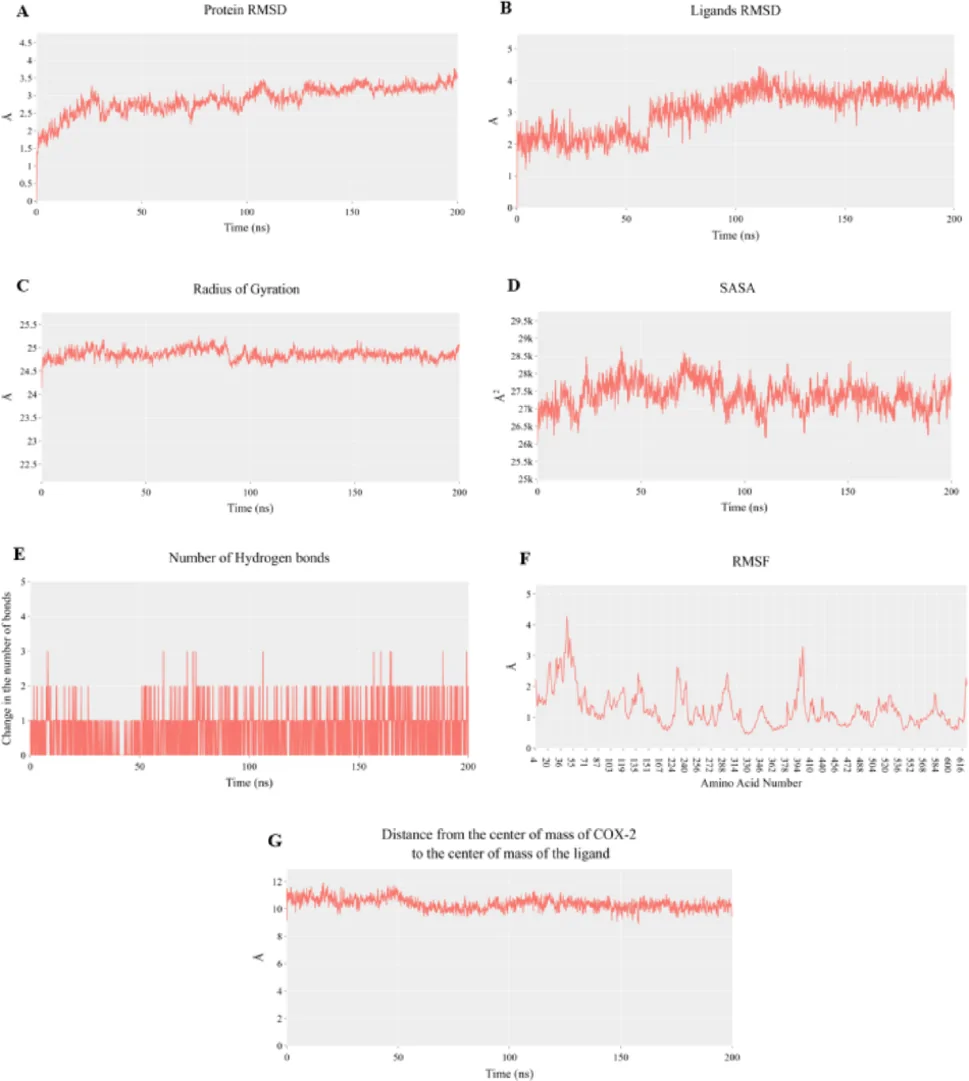
**A** RMSD values from the trajectory for the enzyme COX-2 with compound **7a**, **B** The ligand RMSD values, **C** radius of gyration for the Enzyme COX-2 protein, **D** SASA for enzyme COX-2, **E** Change in the number of hydrogen bonds between compound **7a** and enzyme COX-2, **F** RMSF for enzyme COX-2 protein, **G** Distance from the center of mass of compound **7a** and enzyme COX-2

For compound **7a**, the same analyses were performed. Figure 13A shows the RMSD of COX-2 in complex with **7a**. It shows an increasing trend during the first 25 ns before stabilizing around 3 Å which is similar to the first compound system. For the ligand RMSD (Fig. 13B), the RMSD shows two averages. The first one lasts for around 60 ns with an average around 2 Å before increasing to 3.5 Å for the second half of the simulation. The overall structure of the protein shows a stable solvent accessible surface area (SASA) and radius of gyration (RoG) (Fig. 13C and D) with averages around 27,250 Å^2^ and 24.7 Å, respectively. The ligand was found to for a dominant one stable hydrogen bond during nearly all the simulation time with some frames showing two and three Hbonds interactions (Fig. 13E). The RMSF of the protein (Fig. 13F) shows some regions with an average fluctuation higher than 2 Å which corresponds to the unstructured loops in the protein. Finally, the separation between the centers of mass of the protein and the ligand shows a stable binding with an average value of 10 Å. Overall the simulation of 7a compound with COX-2 shows a stable binding with the protein and stable protein conformation.

**Fig. 14:**
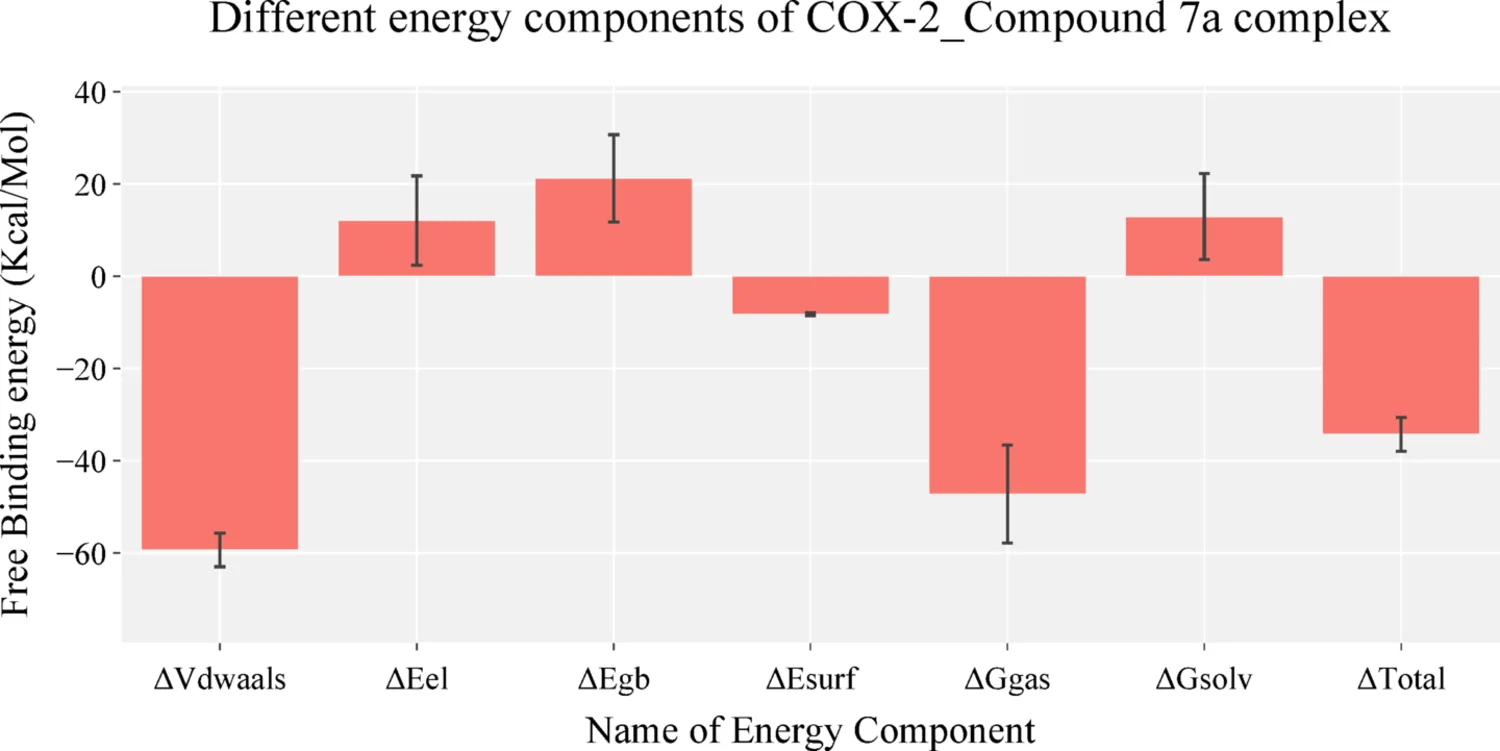
The different energetic components of MM-GBSA and their values for the system. Bars represent the standard deviations

**Fig. 15:**
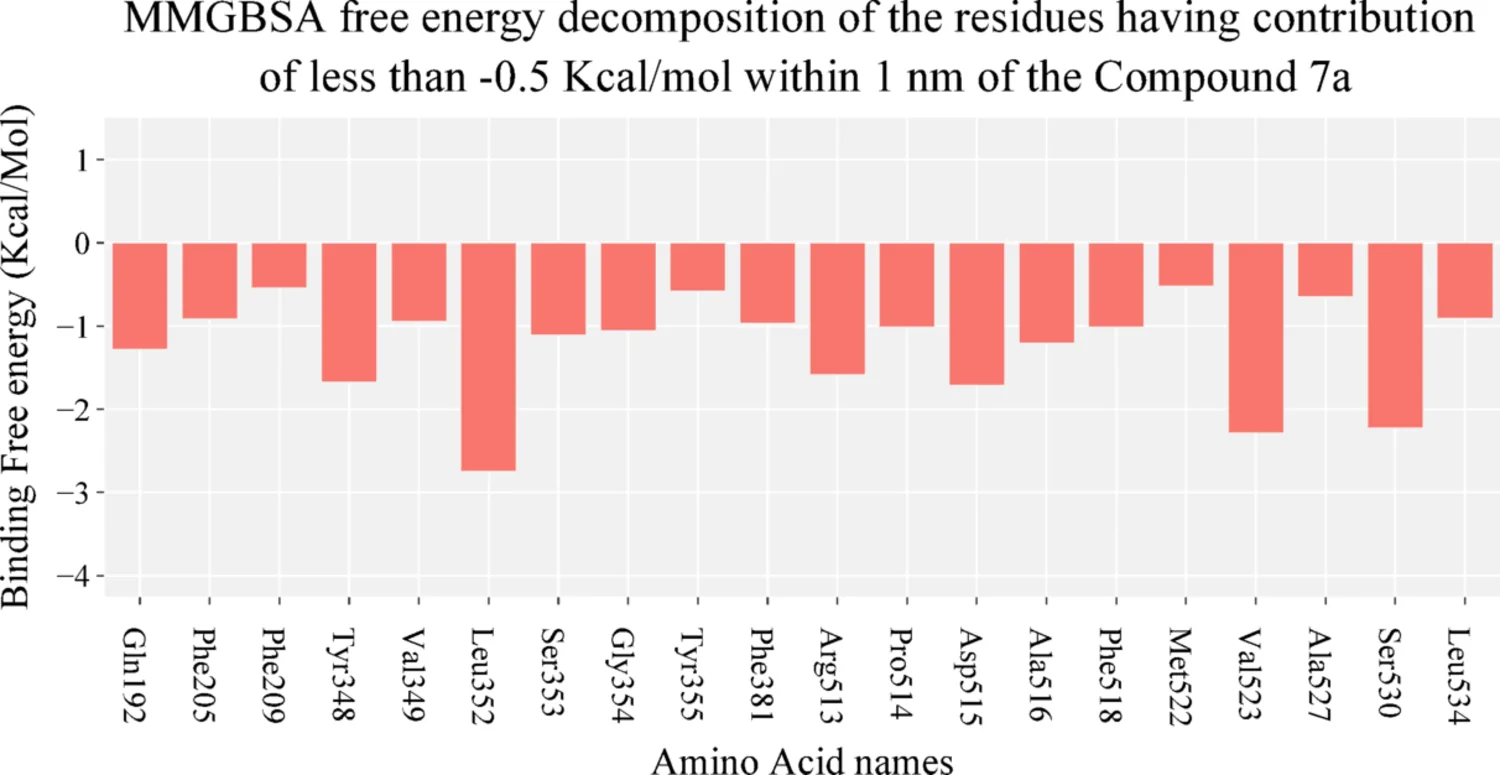
The binding free energy decomposition of the enzyme COX-2_compound **7a** complex. Amino acids shown are the ones within 1 nm of the ligand, and having a binding affinity of -0.5 Kcal/mol or better

To determine the binding free energy, MM-GBSA was used (Fig. 14). The most dominant type of interaction was the van der Waals interaction with an average contribution of -59.33 Kcal/mol while the electrostatic contribution was unfavourable with an average value of + 12.1 Kcal/mol. For the solvation energy, the polar part was unfavourable with an average value of + 21.22 Kcal/mol while the non-polar part had a better contribution with an average of -8.26 Kcal/mol. Overall, the binding energy shows an average of -34.27 Kcal/mol which is slightly better than comoound **4c** which has an average of -31.42 Kcal/mol. Moreover, decomposition analysis was performed to determine the contribution of each amino acid around the ligand (Fig. 15). 12 amino acids were found to have a binding affinity of better than − 1 Kal/mol namely; Gln192 (-1.27 Kcal/mol), Tyr348 (-1.67 Kcal/mol), Leu352 (-2.74 Kcal/mol), Ser353 (-1.11 Kcal/mol), Gly354 (-1.05 Kcal/mol), Arg513 (-1.58 Kcal/mol), Pro514 (-1.01 Kcal/mol), Asp515 (-1.71 Kcal/mol), Ala516 (-1.2 Kcal/mol), Phe518 (-1.01 Kcal/mol), Val523 (-2.28 Kcal/mol), and Ser530 (-2.22 Kcal/mol).

**Fig. 16:**
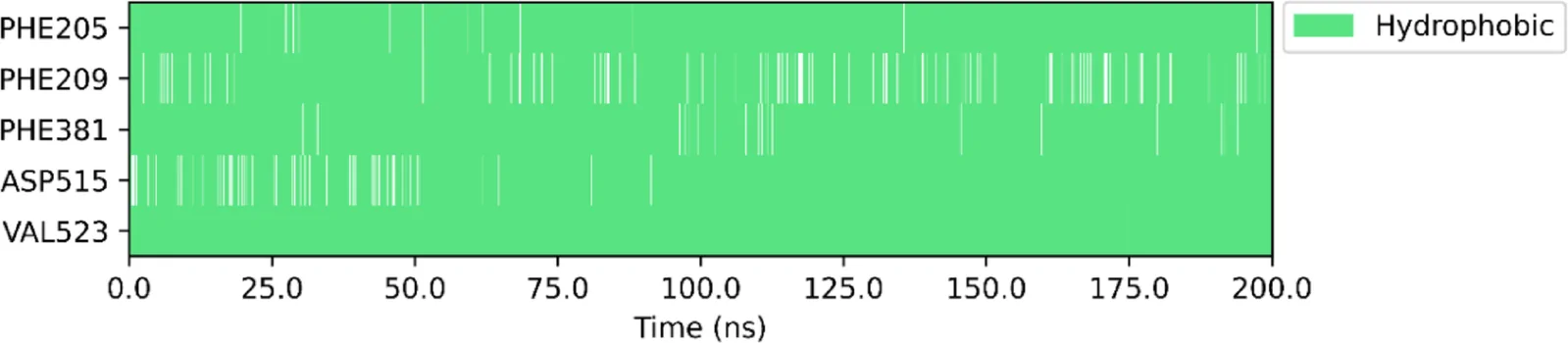
The amino acids, the types of interactions with the compound **7a**, and their occurrence during the whole simulation time using the ProLIF Python library

ProLIF was used to detect the occurrence of different interactions during the run with at least 90% occurrence (Fig. 16) which resulted in five amino acids namely, Phe205 (Hydrophobic interactions 98.7%), Phe209 (Hydrophobic interactions 92.4%), Phe381 (Hydrophobic interactions 98.5%), Asp515 (Hydrophobic interactions 96%), and Val523 (Hydrophobic interactions 99.9%). This indicates the important role of hydrophobic interactions in stabilizing the interaction as shown in the binding energy components (Fig. 14).

### Molecular dynamic simulations on 5-LOX

**Fig. 17:**
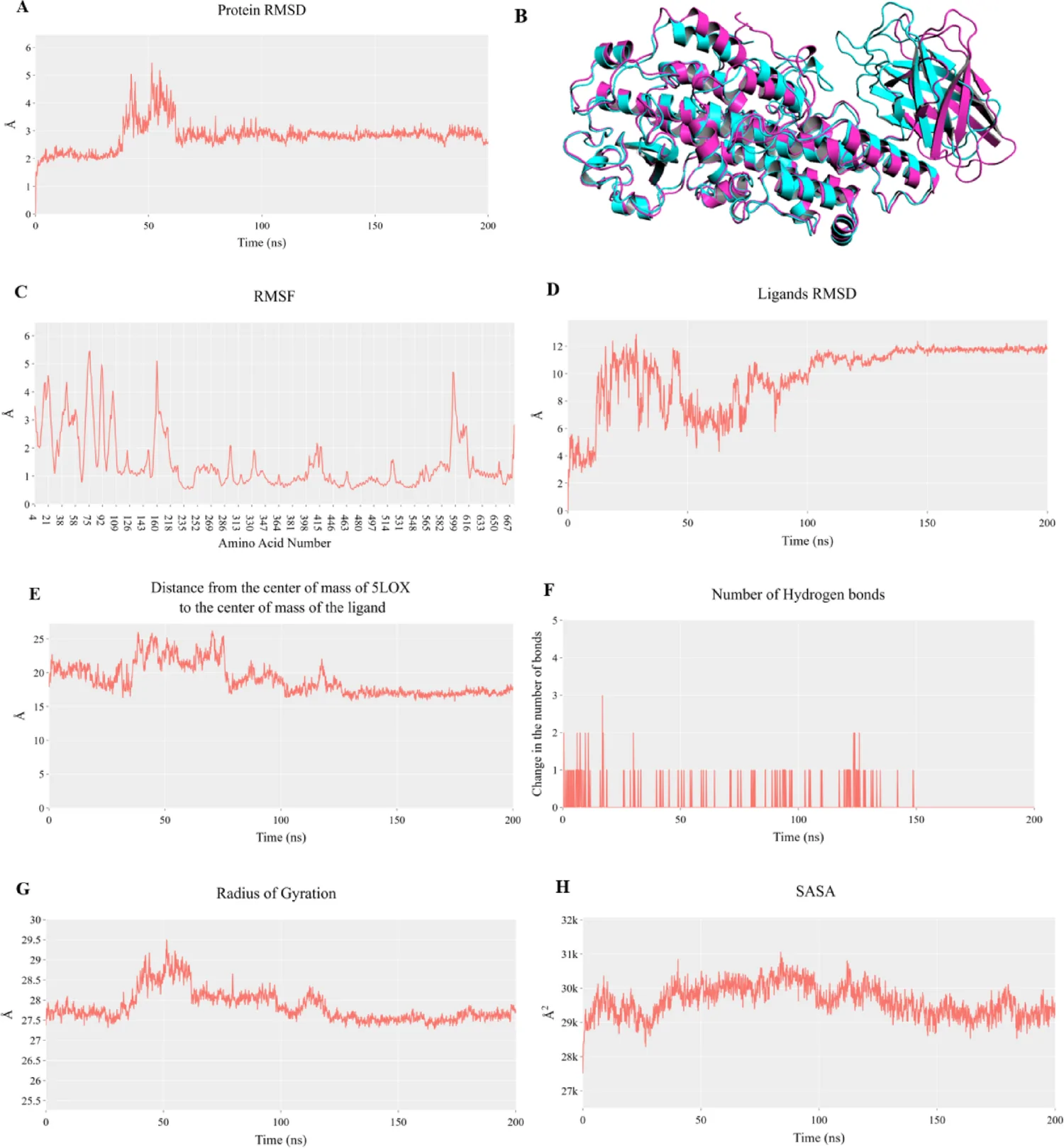
**A** RMSD values from the trajectory for the 5-LOX enzyme, **B** A comparison between the conformation of the protein at 0 ns (cyan cartoon) and its conformation at 51 ns (magenta cartoon) indicating the reason of the large fluctuation around 50 ns, **C** RMSF for 5-LOX enzyme, **D** The ligand RMSD values, **E** The change in the distance from the center of mass of compound **4c** and 5-LOX enzyme, **F** The change in the number of hydrogen bonds between compound **4c** and 5-LOX, **G** The change in the radius of gyration for 5-LOX enzyme, **H** The change in the SASA for 5-LOX enzyme

For 5-LOX enzyme, the same analyses were performed. The protein RMSD shows stable trend for the first 38 ns with an average around 2 Å before transiently increasing to around 4 Å then stabilizing gain around 3 Å (Fig. 17A). this increase was due to the movement of the N-terminal domain as shown in Fig. 17B which compares the structure of the protein at 0 ns (cyan cartoon) with its conformation at 51 ns (magenta cartoon). Moreover, Fig. 17C shows the RMSF of the protein C-α atoms, which shows a high fluctuation in the N-domain (Pro4:Val216). On the other hand, compound **4c** shows a large fluctuation in the first half of the simulation before stabilizing in the second half around an average of 11 Å (Fig. 17D). Similarly, the separation between the center of mass of the protein and that of the ligand showed a similar trend with a large fluctuations during the first half before stabilizing in the second half at 17 Å (Fig. 17E). This change in the conformation of the ligand affected the number of H-bonds formed between the ligand and the protein. During the first 150 ns, at least one H-bond was formed between both of them, while during the last 50 ns, no H-bonds were detected (Fig. 17F). The RoG shows a similar trend to the protein RMSD, with an increase around the 50 ns with an average of 27.5 Å (Fig. 17G). Similarly, the SASA (Fig. 17H) values show a similar trend, increasing around 50 ns.

**Fig. 18:**
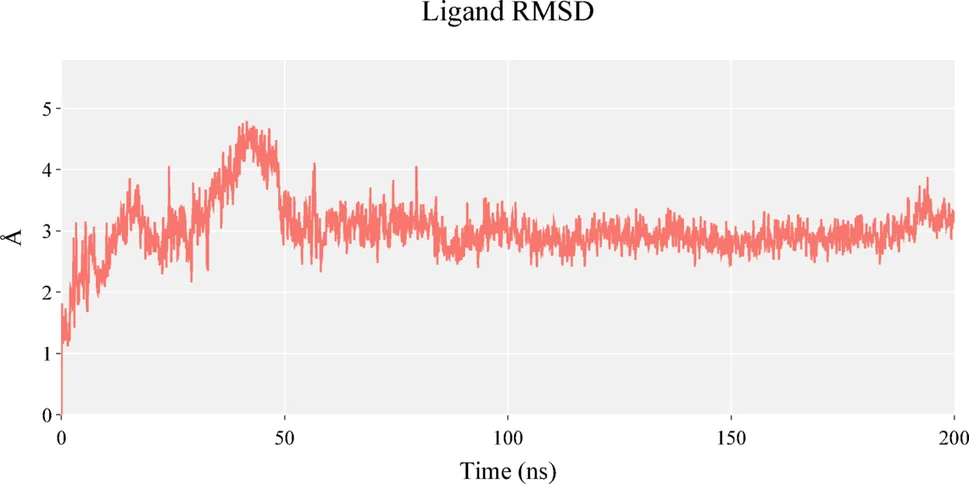
The different energetic components of MM-GBSA and their values for the system. Bars represent the standard deviations

**Fig. 19:**
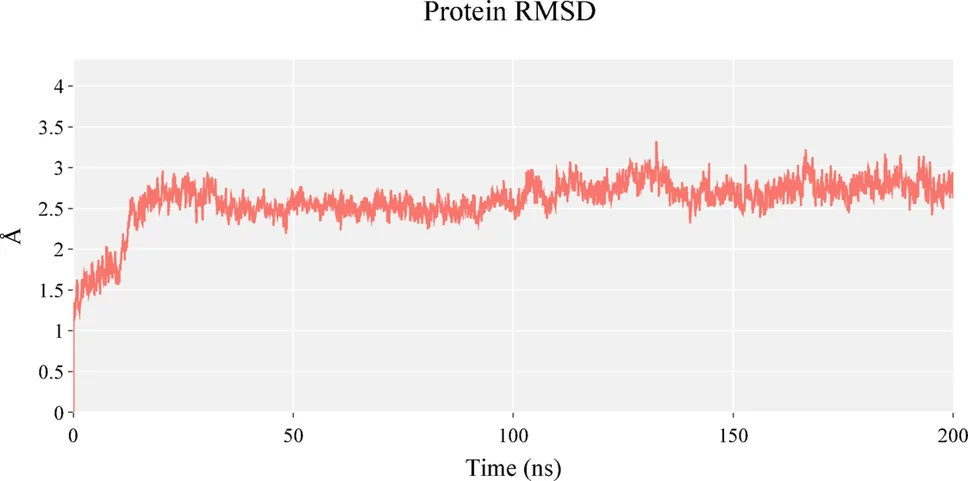
The binding free energy decomposition of the 5-LOX_compound **4c** complex. Amino acids shown are within 1 nm of the ligand, and have a binding affinity of -0.5 Kcal/mol or better

**Fig. 20:**
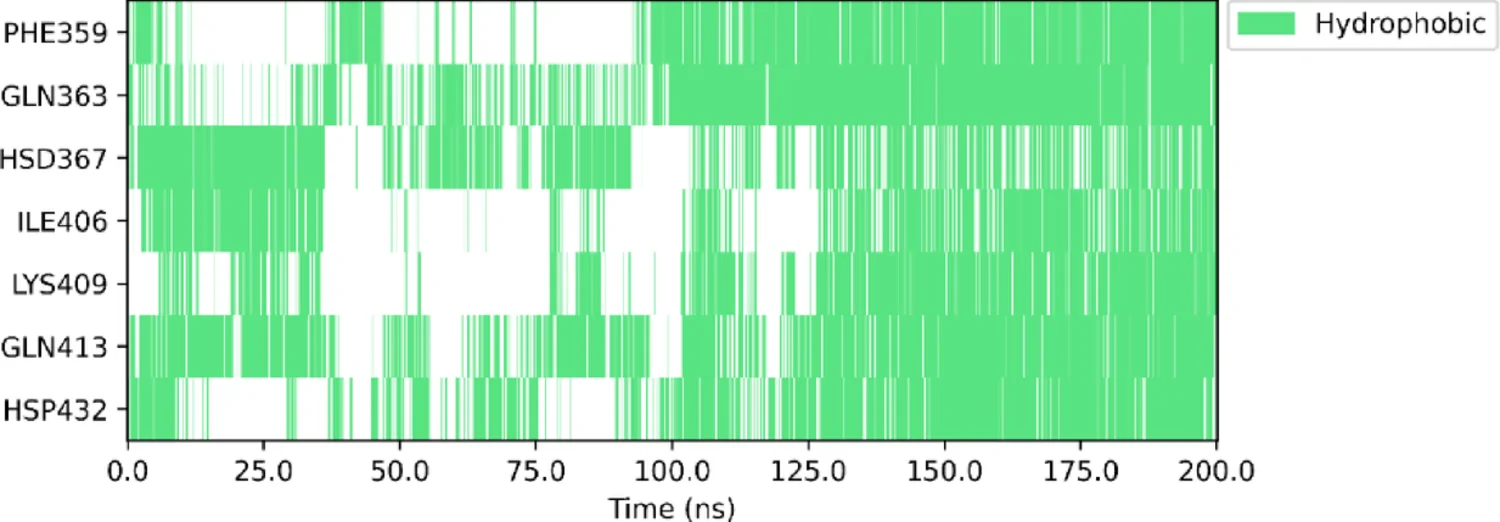
The amino acids, the interactions with compound **4c**, and their occurrence during the simulation time using the ProLIF Python library

To determine the binding strength between the 5-LOX and compound **4c**, we performed MM-GBSA calculations, which yielded an average binding energy of -13.45 Kcal/mol (Fig. 18). Its favourable energetic components are van der Waals interaction with an average of -34.57 Kcal/mol, electrostatic interactions with an average of -13.42 Kcal/mol and non-polar solvation energy of -5.04 Kcal/mol. On the other hand, the polar solvation energy had a significant non-favourable value of + 39.58 Kcal/mol. To determine which amino acids are involved in the interaction with the protein, amino acid decomposition was performed. Only two amino acids were found to form an interaction with a contribution of better than − 1.0 Kcal/mol (Gln363: -1.3 Kcal/mol and Gln413: -1.22 Kcal/mol) (Fig. 19). Non-covalent interactions were further quantified with ProLIF, and two amino acids were found to form hydrophobic interactions with a proportion of 72% (Gln363) and 75% (Gln413) of the simulation time (Fig. 20). The rest of the amino acids were found to form hydrophobic interactions with compound **4c**.

## Conclusion

In this study two new sets of phthalimide derivatives **4a-h** and **7a-f** were designed and synthesized to discover effective anti-inflammatory agents with dual COX-2/5-LOX inhibition activity. To avoid the drawbacks of both highly selective and non-selective COX-2 inhibitors, the compounds in this study were designed to be moderately selective COX-2 inhibitors. Also, the ability of the target compounds to inhibit 5-LOX enzyme was assessed because it is one of the main reasons of the bronchial asthma and other inflammatory diseases. The findings of the in vitro screening demonostrated that the compounds under investigation have lower inhibitory activity toward COX-1 compared to indomethacin, indicating better safety profile. Compounds **4a**, **4c**, **4f**, **7a**, **7b** and **7f** showed better inhibitory effect against COX-2 [IC_50_ = 0.99–6.28 µM] compared to the reference drug meloxicam [IC_50_ = 8.34 µM]. Compounds **4c** and **7a** are the most potent ones on COX-2 with IC_50_ less than 1 µM and moderate selectivity indices = 7.6 and 5.6, respectively which less than that of celecoxib but greater than meloxicam. Also, the two compounds have potent 5-LOX inhibition effect with IC_50_ values comparable to that of zileuton. Also, all of the tested compounds could suppress the nitric oxide production in LPS-induced RAW 264.7 macrophages at 100 µM. Furthermore, compounds **4c** and **7a** caused a reduction of the expression of the inflammatory mediators PGE-2, IL-6 and TNF-α supporting their anti-inflammatory effect in macrophages. Finally, molecular docking studies as well molecular dynamic simulations were carried out to investigate the binding intercations of of the most active compounds into the active site of both COX-2 and 5-LOX. Accordingly, the achieved results of this work could pave the way toward further development of phthalimide-based compounds as new eff anti-inflammatory drugs.

### Experimental

#### Chemistry

##### General

Melting points were determined with Electrothermal Capillary melting point apparatus and were uncorrected. The IR spectra were recorded on Shimadzu IR 435 spectrophotometer, and values were represented in cm^-1^. ^1^H and ^13^C NMR spectra were recorded on 400 (100) MHz Bruker or 500 (125) MHz Jeol spectrometers using tetramethylsilane (TMS) as an internal standard. Mass spectra were carried on Thermo Scientific ISQ single quadrupole mass spectrometer. High resolution mass spectra were recorded on Q-TOF, 6530 (Agilent Technologies). Elemental analyses were performed on FLASH 2000 CHNS/O analyzer, Thermo Scientific (USA). For thin layer chromatography (TLC), Silica gel F254 plates (E. MERCK) were used and visualization was performed by illumination with UV-light source (254 nm).

### General procedure for the synthesis of Methyl 4-(2-(substituted)acetamido) benzoate 2a-h and 5a-f

A mixture of methyl 4-(2-chloroacetamido)benzoate (**1**) (2.27 gm,10 mmol) and NaI (1.49 gm, 10 mmol) in 20 ml acetone was heated at 56 °C for one hour followed by addition of 10 mmol of the appropriate amine and anhydrous sodium carbonate (1 gm,10 mmol). The mixture was further refluxed for 3 h. After cooling to room temperature, the mixture was filtered and the filtrate was evaporated under reduced pressure. The residue was washed with 20 ml water and extracted three times with 30 ml ethyl acetate. The organic layer was dried and evaporated to get compounds **2a-h** and **5a-f** which were further purified by column chromatography using silica gel as a stationary phase and a mixture of n-hexane: ethyl acetate 7:3 as a mobile phase.

**Methyl 4-(2-(dimethylamino)acetamido)benzoate** (**2a**) Yield 66%; yellow solid; Rf = 0.7; m.p. 63 °C; IR (KBr, cm^− 1^): 3298 (NH), 1716 (C = O), 1681 (C = O); ^1^H-NMR (400 MHz, CDCl_3_) *δ* ppm 2.41 (s, 6 H, N-(CH_3_)_2_), 3.12 (s, 2 H, CH_2_-C = O), 3.88 (s, 3 H, CH_3_), 7.67 (d, 2 H, Ar-H, *J* = 8.8 Hz), 8.0 (d, 2 H, Ar-H, *J* = 8.8 Hz), 9.41 (s, 1H, NH); ^13^C-NMR (100 MHz, CDCl_3_) *δ* ppm 45.8, 51.8, 63.3, 118.3, 125.2, 130.6, 141.8, 166.5, 168.8; HRMS (ESI) calculated for C_12_H_16_N_2_O_3_ [M + H]^+^: 237.12392, found 237.12444.

**Methyl 4-(2-(diethylamino)acetamido)benzoate** (**2b**) Yield 84%; yellow solid; Rf = 0.7; m.p. 60 °C reported m.p. 60–62 °C [66]; ^1^H-NMR (500 MHz, CDCl_3_) *δ* ppm 1.09 (t, 6 H, (CH_3_)_2_, *J* = 10 Hz), 2.68 (brs, 4 H, (CH_2_)_2_), 3.23 (s, 2 H, CH_2_-C = O), 3.89 (s, 3 H, CH_3_), 7.65 (d, 2 H, Ar-H, *J* = 10 Hz), 7.99 (d, 2 H, Ar-H, *J* = 10 Hz), 9.61 (s, 1H, NH); ^13^C-NMR (125 MHz, CDCl_3_) *δ* ppm 12.4, 48.9, 52.0, 58.0, 118.5, 125.6, 130.4, 141.4, 166.6, 169.9.

**Methyl 4-(2-(dipropylamino)acetamido)benzoate** (**2c**) Yield 80%; yellow solid; Rf = 0.7; m.p. 44 °C; IR (KBr, cm^− 1^): 3309 (NH), 1712 (C = O), 1678 (C = O); ^1^H-NMR (400 MHz, CDCl_3_) *δ* ppm 0.93 (t, 6 H, *J* = 7.2 Hz, (CH_3_)_2_), 1.51–1.54 (m, 4 H, (*CH*_*2*_-CH_3_)_2_), 2.52 (t, 4 H, *J* = 7.2 Hz, (*CH*_*2*_-CH_2_-CH_3_)_2_), 3.19 (s, 2 H, CH_2_-C = O), 3.88 (s, 3 H, CH_3_), 7.64 (d, 2 H, Ar-H, *J* = 8.8 Hz), 8.01 (d, 2 H, Ar-H, *J* = 8.4 Hz), 9.65 (s, 1H, NH); ^13^C-NMR (100 MHz, CDCl_3_) *δ* ppm 11.8, 20.4, 51.8, 57.2, 59.5, 118.1, 125.0, 130.5, 141.8, 166.6, 170.2; HRMS (ESI) calculated for C_16_H_24_N_2_O_3_ [M + H]^+^: 293.18652, found 293.18915.

**Methyl 4-(2-(diisopropylamino)acetamido)benzoate** (**2d**) Yield 79%; buff solid; Rf = 0.7; m.p. 100 °C; IR (KBr, cm^− 1^): 3267 (NH), 1716 (C = O), 1685 (C = O); ^1^H-NMR (400 MHz, CDCl_3_) *δ* 0.90 (d, 12 H, *J* = 6 Hz, (CH(*CH*_*3*_)_2_)_2_), 2.91-3.00 (m, 4 H, CH_2_-C = O, (*CH*(CH_3_)_2_)_2_), 3.72 (s, 3 H, CH_3_), 7.46 (d, 2 H, Ar-H, *J* = 8 Hz), 7.83 (d, 2 H, Ar-H, *J* = 8 Hz), 9.52 (s, 1H, NH); ^13^C-NMR (100 MHz, CDCl_3_) *δ* 20.7, 50.0, 52.2, 118.3, 125.0, 130.9, 141.8, 166.2, 172.0; HRMS (ESI) calculated for C_16_H_24_N_2_O_3_ [M + H]^+^: 293.18652, found 293.18750.

**Methyl 4-(2-(piperidin-1-yl)acetamido)benzoate** (**2e**) Yield 69%; white solid; Rf = 0.6; m.p. 132 °C; IR (KBr, cm^− 1^): 3298 (NH), 1712 (C = O), 1681 (C = O); ^1^H-NMR (400 MHz, CDCl_3_) *δ* 1.50–1.51 (m, 2 H, CH_2_), 1.63–1.69 (m, 4 H, (CH_2_)_2_), 2.55 (brs, 4 H, (CH_2_)_2_), 3.09 (s, 2 H, CH_2_-C = O), 3.90 (s, 3 H, CH_3_), 7.65 (d, 2 H, Ar-H, *J* = 8.8 Hz), 8.01 (d, 2 H, Ar-H, *J* = 8.8 Hz), 9.50 (s, 1H, NH); ^13^C-NMR (100 MHz, CDCl_3_) *δ* 23.4, 26.0, 51.8, 55.1, 62.7, 118.1, 125.1, 130.5, 141.8, 166.6, 169.5; HRMS (ESI) calculated for C_15_H_20_N_2_O_3_ [M + H]^+^: 277.15522, found 277.15685.

**Methyl 4-(2-morpholinoacetamido)benzoate** (**2f**) Yield 75%; yellow solid; Rf = 0.6; m.p. 112 °C reported m.p. 113–114 °C [66]. ^1^H-NMR (500 MHz, CDCl_3_) δ ppm 2.65 (brs, 4 H, (CH_2_)_2_), 3.18 (s, 2 H, CH_2_-C = O), 3.77 (brs, 4 H, (CH_2_)_2_, 3.88 (s, 3 H, CH_3_), 7.63 (d, 2 H, Ar-H, *J* = 10 Hz), 7.99 (d, 2 H, Ar-H, *J* = 10 Hz), 9.30 (s, 1H, NH); ^13^C-NMR (125 MHz, CDCl_3_) *δ* ppm 52.0, 53.8, 62.4, 67.0, 118.6, 125.8, 130.9, 141.6, 166.6, 168.2.

**Methyl 4-(2-(3**,**4-dihydroisoquinolin-2(1*H*)-yl)acetamido)benzoate** (**2 g**) Yield 88%; yellow solid; Rf = 0.4; m.p. 108 °C; IR (KBr, cm^− 1^): 3267 (NH), 1708 (C = O), 1693 (C = O); ^1^H-NMR (400 MHz, CDCl_3_) *δ* 3.00-3.04 (m, 4 H, CH_2_-CH_2_-N), 3.43 (s, 2 H, CH_2_-C = O), 3.90–3.91 (m, 5 H, CH_3_, CH_2_-N), 7.05 (d, 1H, Ar-H, *J* = 7.2 Hz), 7.18–7.23 (m, 3 H, Ar-H), 7.67 (d, 2 H, Ar-H, *J* = 8.8 Hz), 8.01 (d, 2 H, Ar-H, *J* = 8.8 Hz), 9.56 (s, 1H, NH); ^13^C-NMR (100 MHz, CDCl_3_) *δ* 28.9, 51.4, 52.0, 55.8, 61.7, 118.7, 125.6, 126.2, 126.5, 126.8, 128.8, 130.8, 133.1, 141.6, 166.5, 168.0; HRMS (ESI) calculated for C_19_H_20_N_2_O_3_ [M + H]^+^: 325.15522, found 325.15742.

**Methyl 4-(2-(4-phenylpiperazin-1-yl)acetamido)benzoate** (**2 h**) Yield 65%; white solid; Rf = 0.5; m.p. 154 °C; IR (KBr, cm^− 1^): 3294 (NH), 1720 (C = O), 1685 (C = O); ^1^H-NMR (500 MHz, CDCl_3_) *δ* ppm 2.84 (brs, 4 H, (CH_2_)_2_), 3.29 (brs, 6 H, CH_2_-C = O, (CH_2_)_2_), 3.89 (s, 3 H, CH_3_), 6.93–6.95 (m, 3 H, Ar-H), 7.27–7.30 (m, 2 H, Ar-H), 7.65 (d, 2 H, Ar-H, *J* = 8.5 Hz), 8.01 (d, 2 H, Ar-H, *J* = 8.6 Hz), 9.42 (s, 1H, NH); ^13^C-NMR (125 MHz, CDCl_3_) *δ* 49.3, 52.0, 53.6, 62.0, 116.4, 118.6, 120.4, 125.8, 129.3, 130.9, 141.7, 150.9, 166.6, 169.7; HRMS (ESI) calculated for C_20_H_23_N_3_O_3_ [M + H]^+^: 354.18177, found 354.18323.

**Methyl 4-(2-(4-(phenylsulfonyl)piperazin-1-yl)acetamido)benzoate** (**5a**) Yield 60%; buff solid; Rf = 0.45; m.p. 172 °C; IR (KBr, cm^− 1^): 3290 (NH), 1708 (C = O), 1685 (C = O); ^1^H-NMR (400 MHz, CDCl_3_) *δ* 2.74 (brs, 4 H, (CH_2_)_2_), 3.15 (d, 6 H, CH_2_-C = O, (CH_2_)_2_, *J* = 16 Hz), 3.89 (s, 3 H, CH_3_), 7.55–7.69 (m, 5 H, Ar-H), 7.78 (d, 2 H, Ar-H, *J* = 7.6 Hz), 7.96 (d, 2 H, Ar-H, *J* = 8.8 Hz), 8.94 (s, 1H, NH); ^13^C-NMR (100 MHz, CDCl_3_) *δ* 45.7, 52.0, 52.6, 61.5, 118.7, 125.8, 127.7, 129.2, 130.8, 133.0, 135.7, 141.3, 166.5, 166.9; HRMS (ESI) calculated for C_20_H_23_N_3_O_5_S [M + H]^+^: 418.14367, found 418.14457.

**Methyl 4-(2-(4-((4-chlorophenyl)sulfonyl)piperazin-1-yl)acetamido)benzoate** (**5b**) Yield 65%; Rf = 0.4; yellow solid; m.p. 176 °C; IR (KBr, cm^− 1^): 3275 (NH), 1708 (C = O), 1680 (C = O); ^1^H-NMR (400 MHz, CDCl_3_) *δ* 2.74 (t, 4 H, (CH_2_)_2_, *J* = 9.2 Hz), 3.15 (brs, 4 H, (CH_2_)_2_), 3.21 (s, 2 H, CH_2_-C = O), 3.90 (s, 3 H, CH_3_), 7.56–7.59 (m, 4 H, Ar-H), 7.72 (d, 2 H, Ar-H, *J* = 8.4 Hz), 7.98 (d, 2 H, Ar-H, *J* = 8.4 Hz), 8.89 (s, 1H, NH); ^13^C-NMR (100 MHz, CDCl_3_) *δ* 45.7, 52.0, 52.6, 61.6, 118.7, 125.9, 129.1, 129.6, 130.8, 134.2, 139.8, 141.2, 166.4, 167.4; HRMS (ESI) calculated for C_20_H_22_ClN_3_O_5_S [M + H]^+^: 452.10470, found 452.10610.

**Methyl 4-(2-(4-((4-bromophenyl)sulfonyl)piperazin-1-yl)acetamido)benzoate** (**5c**) Yield 61%; yellow solid; Rf = 0.4; m.p. 164 °C; IR (KBr, cm^− 1^): 3251 (NH), 1708 (C = O), 1678 (C = O); ^1^H-NMR (400 MHz, DMSO-*d*_*6*_) *δ* 2.60 (brs, 4 H, (CH_2_)_2_), 2.99 (brs, 4 H, (CH_2_)_2_), 3.19 (s, 2 H, CH_2_-C = O), 3.81 (s, 3 H, CH_3_), 7.67–7.75 (m, 4 H, Ar-H), 7.87–7.90 (m, 4 H, Ar-H), 9.93 (s, 1H, NH); ^13^C-NMR (100 MHz, DMSO-*d*_*6*_) *δ* 45.3, 51.5, 51.8, 60.8, 119.0, 124.1, 127.3, 129.5, 130.0, 132.5, 134.2, 142.8, 165.6, 168.4; HRMS (ESI) calculated for C_20_H_22_BrN_3_O_5_S [M + H]^+^: 496.05418, found 496.05736.

**Methyl 4-(2-(4-tosylpiperazin-1-yl)acetamido)benzoate** (**5d**) Yield 68%; white solid; Rf = 0.45; m.p. 182 °C; IR (KBr, cm^− 1^): 3294 (NH), 1712 (C = O), 1697 (C = O); ^1^H-NMR (400 MHz, CDCl_3_) *δ* 2.48 (s, 3 H, CH_3_) 2.72 (t, 4 H, (CH_2_)_2_, *J* = 9.2 Hz), 3.14 (brs, 4 H, (CH_2_)_2_), 3.20 (s, 2 H, CH_2_-C = O), 3.89 (s, 3 H, CH_3_), 7.37 (d, 2 H, Ar-H, *J* = 8 Hz), 7.55 (d, 2 H, Ar-H, *J* = 8.4 Hz), 7.66 (d, 2 H, Ar-H, *J* = 8.4 Hz), 7.97 (d, 2 H, Ar-H, *J* = 8.8 Hz), 8.94 (s, 1H, NH); ^13^C-NMR (100 MHz, CDCl_3_) *δ* 21.2, 45.7, 52.0, 52.6, 61.3, 118.7, 125.8, 127.8, 129.9, 130.7, 132.6, 141.3, 144.0, 166.4, 167.5; HRMS (ESI) calculated for C_21_H_25_N_3_O_5_S [M + H]^+^: 432.15932, found 432.16172.

**Methyl 4-(2-(4-((4-methoxyphenyl)sulfonyl)piperazin-1-yl)acetamido)benzoate** (**5e**) Yield 70%; buff solid; Rf = 0.45; m.p. 158 °C; IR (KBr, cm^− 1^): 3282 (NH), 1712 (C = O), 1693 (C = O); ^1^H-NMR (400 MHz, CDCl_3_) *δ* 2.74 (brs, 4 H, (CH_2_)_2_), 3.12 (d, 6 H, CH_2_-C = O, (CH_2_)_2_, *J* = 16 Hz), 3.88 (d, 6 H, CH_3_, OCH_3_, *J* = 8 Hz), 7.02 (d, 2 H, Ar-H, *J* = 8.4 Hz), 7.55 (d, 2 H, Ar-H, *J* = 8.4 Hz), 7.70 (d, 2 H, Ar-H, *J* = 8.4 Hz), 7.95 (d, 2 H, Ar-H, *J* = 8.4 Hz), 8.98 (s, 1H, NH); ^13^C-NMR (100 MHz, CDCl_3_) *δ* 45.5, 51.8, 52.4, 55.5, 61.4, 114.2, 118.5, 125.6, 126.9, 129.7, 130.6, 141.1, 163.1, 166.3, 167.2; HRMS (ESI) calculated for C_21_H_25_N_3_O_6_S [M + H]^+^: 448.15424, found 448.15761.

**Methyl 4-(2-(4-((4-(trifluoromethyl)phenyl)sulfonyl)piperazin-1-yl)acetamido)benzoate** (**5f**) Yield 64%; white solid; Rf = 0.45; m.p. 166 °C; IR (KBr, cm^− 1^): 3259 (NH), 1705 (C = O), 1693 (C = O); ^1^H-NMR (400 MHz, CDCl_3_) *δ* 2.69 (t, 4 H, (CH_2_)_2_, *J* = 8 Hz), 3.17 (brs, 6 H, CH_2_-C = O, (CH_2_)_2_), 3.86 (s, 3 H, CH_3_), 7.52 (d, 2 H, Ar-H, *J* = 8.8 Hz), 7.81–7.95 (m, 6 H, Ar-H), 8.85 (s, 1H, NH); ^13^C-NMR (100 MHz, CDCl_3_) *δ* 45.6, 51.9, 52.4, 61.5, 118.6, 125.7, 126.3 (d, *J* = 6.25 Hz), 128.0, 130.6, 134.3, 134.8, 139.4, 141.1, 166.3, 167.4; HRMS (ESI) calculated for C_21_H_22_F_3_N_3_O_5_S [M + H]^+^: 486.13106, found 486.13317.

**General procedure for the synthesis of** ***N*-(4-(hydrazinecarbonyl)phenyl)-2-(substituted)acetamide 3a-h and 6a-f**.

A mixture of methyl ester **2a-h** or **5a-f** (5 mmol) and hydrazine hydrate (25 mmol) in 5 ml of methanol was refluxed for 4 h. After cooling to room temperture a white precipitate of the desired acid hydrazide **3a-h** or **6a-f** was formed and filtered off. The product was re-crystallized from ethanol to give the compound in a pure form.

**2-(Dimethylamino)-*N*-(4-(hydrazinecarbonyl)phenyl)acetamide** (**3a**) Yield 60%; white solid; m.p. 120 °C; IR (KBr): 3321, 3260 (NH_2_, NH), 1685, 1624 (C = O); ^1^H-NMR (400 MHz, DMSO-*d*_*6*_) *δ* 2.27 (s, 6 H, N-(CH_3_)_2_), 3.09 (s, 2 H, CH_2_-C = O), 4.03 (brs, 2 H, NH_2_), 7.71 (d, 2 H, Ar-H, *J* = 8.8 Hz), 7.77 (d, 2 H, Ar-H, *J* = 8.8 Hz), 9.65 (s, 1H, NH), 9.92 (s, 1H, NH); ^13^C-NMR (100 MHz, DMSO-*d*_*6*_) *δ* 45.7, 63.7, 119.1, 128.1, 128.3, 141.6, 166.2, 169.5; Ms m/z (EI) 236.12 (M^+^) (77%); Anal. Calcd for C_11_H_16_N_4_O_2_: C, 55.92; H, 6.83; N, 23.71. Found: C, 56.18; H, 7.02; N, 23.95.

**2-(Diethylamino)-*N*-(4-(hydrazinecarbonyl)phenyl)acetamide** (**3b**) Yield 75%; white solid; m.p. 115 °C; IR (KBr): 3325, 3267 (NH_2_, NH), 1670, 1624 (C = O); ^1^H-NMR (400 MHz, DMSO-*d*_*6*_) *δ* 0.99 (t, 6 H, (CH_3_)_2_, *J* = 7.2 Hz), 2.57 (q, 4 H, (CH_2_)_2_, *J* = 7.2 Hz) 3.17 (s, 2 H, CH_2_-C = O), 7.70 (d, 2 H, Ar-H, *J* = 8.4 Hz), 7.78 (d, 2 H, Ar-H, *J* = 8.4 Hz), 9.67 (s, 1H, NH), 9.84 (s, 1H, NH); ^13^C-NMR (100 MHz, DMSO-*d*_*6*_) *δ* 12.5, 48.2, 57.7, 119.2, 128.3, 128.4, 141.1, 166.2, 170.9; Ms m/z (EI) 264.17 (M^+^) (7.6%); Anal. Calcd for C_13_H_20_N_4_O_2_: C, 59.07; H, 7.63; N, 21.20. Found: C, 59.30; H, 7.78; N, 21.47.

**2-(Dipropylamino)-*N*-(4-(hydrazinecarbonyl)phenyl)acetamide** (**3c**) Yield 70%; white solid; m.p. 80 °C; IR (KBr): 3313, 3267 (NH_2_, NH), 1666, 1620 (C = O); ^1^H-NMR (400 MHz, DMSO-*d*_*6*_) *δ* 0.85 (t, 6 H, *J* = 7.6 Hz, (CH_3_)_2_), 1.41–1.50 (m, 4 H, (*CH*_*2*_-CH_3_)_2_), 2.47–2.50 (m, 4 H, (*CH*_*2*_-CH_2_-CH_3_)_2_), 3.19 (s, 2 H, CH_2_-C = O), 4.40 (brs, 2 H, NH_2_), 7.67 (d, 2 H, Ar-H, *J* = 8.8 Hz), 7.78 (d, 2 H, Ar-H, *J* = 8.4 Hz), 9.66 (s, 1H, NH), 9.76 (s, 1H, NH); ^13^C-NMR (100 MHz, DMSO-*d*_*6*_) *δ* 12.1, 20.5, 56.5, 58.7, 118.7, 128.2, 128.4, 141.2, 165.9, 170.2; Ms m/z (EI) 292.61 (M^+^) (6.3%); Anal. Calcd for C_15_H_24_N_4_O_2_: C, 61.62; H, 8.27; N, 19.16. Found: C, 61.49; H, 8.43; N, 19.40.

**2-(Diisopropylamino)-*N*-(4-(hydrazinecarbonyl)phenyl)acetamide** (**3d**) Yield 68%; white solid; m.p. 96 °C; IR (KBr): 3321, 3259 (NH_2_, NH), 1693, 1620 (C = O); ^1^H-NMR (400 MHz, DMSO-*d*_*6*_) *δ* 1.01 (d, 12 H, *J* = 6.8 Hz, (CH(*CH*_*3*_)_2_)_2_), 3.01–3.06 (m, 2 H, (*CH*(CH_3_)_2_)_2_), 3.13 (s, 2 H, CH_2_-C = O), 4.45 (s, 2 H, NH_2_), 7.71 (d, 2 H, Ar-H, *J* = 8.8 Hz), 7.80 (d, 2 H, Ar-H, *J* = 8.8 Hz), 9.67 (s, 1H, NH), 9.71 (s, 1H, NH); ^13^C-NMR (100 MHz, DMSO-*d*_*6*_) *δ* 20.5, 50.20, 50.28, 118.8, 128.2, 128.5, 141.1, 165.9, 172.0; Ms m/z (EI) 292.79 (M^+^) (14%); Anal. Calcd for C_15_H_24_N_4_O_2_: C, 61.62; H, 8.27; N, 19.16. Found: C, 61.75; H, 8.41; N, 19.37.

***N*-(4-(Hydrazinecarbonyl)phenyl)-2-(piperidin-1-yl)acetamide** (**3e**) Yield 60%; white solid; m.p. 185 °C reported m.p.185–186 °C [67]; ^1^H-NMR (500 MHz, DMSO-*d*_*6*_) *δ* ppm 1.36 (brs, 2 H, CH_2_), 1.53 (brs, 4 H, (CH_2_)_2_), 2.42 (brs, 4 H, (CH_2_)_2_), 3.05 (s, 2 H, CH_2_-C = O), 4.41 (s, 2 H, NH_2_), 7.66 (d, 2 H, Ar-H, *J* = 10 Hz), 7.75 (d, 2 H, Ar-H, *J* = 10 Hz), 9.62 (s, 1H, NH), 9.82 (s, 1H, NH); ^13^C-NMR (125 MHz, DMSO-*d*_*6*_) *δ* ppm 24.0, 25.9, 54.5, 63.2, 118.9, 128.2, 128.5, 141.4, 165.7, 169.0.

***N*-(4-(Hydrazinecarbonyl)phenyl)-2-morpholinoacetamide** (**3f**) Yield 68%; white solid; m.p. 178 °C reported melting point 173–174 °C [67]; ^1^H-NMR (500 MHz, DMSO-*d*_*6*_) *δ* ppm 2.47 (brs, 4 H, (CH_2_)_2_), 3.11 (s, 2 H, CH_2_-C = O), 3.59–3.61 (m, 7 H, CH_3_, (CH_2_)_2_), 4.40 (s, 2 H, NH_2_), 7.65 (d, 2 H, Ar-H, *J* = 10 Hz), 7.75 (d, 2 H, Ar-H, *J* = 10 Hz), 9.62 (s, 1H, NH), 9.89 (s, 1H, NH); ^13^C-NMR (125 MHz, DMSO-*d*_*6*_) *δ* ppm 53.6, 62.5, 66.6, 119.4, 128.2, 128.5, 141.7, 166.1, 169.0.

**2-(3**,**4-Dihydroisoquinolin-2(1*H*)-yl)-*N*-(4-(hydrazinecarbonyl)phenyl)acetamide** (**3 g**) Yield 78%; white solid; m.p. 206 °C; IR (KBr): 3302, 3241 (NH_2_, NH), 1678, 1627 (C = O); ^1^H-NMR (400 MHz, DMSO-*d*_*6*_) *δ* 2.80–2.90 (m, 4 H, CH_2_-CH_2_-N), 3.34 (s, 2 H, CH_2_-C = O), 3.73 (s, 2 H, CH_2_-N), 4.44 (s, 2 H, NH_2_), 7.05–7.12 (m, 4 H, Ar-H), 7.71 (d, 2 H, Ar-H, *J* = 8.8 Hz), 7.77 (d, 2 H, Ar-H, *J* = 8.8 Hz), 9.65 (s, 1H, NH), 9.98 (s, 1H, NH); ^13^C-NMR (100 MHz, DMSO-*d*_*6*_) *δ* 28.9, 50.9, 55.5, 62.0, 119.2, 126.0, 126.5, 126.8, 128.1, 128.4, 128.9, 134.3, 134.9, 141.5, 165.9, 169.5; Ms m/z (EI) 323.92 (M^+^) (24.2%); Anal. Calcd for C_18_H_20_N_4_O_2_: C, 66.65; H, 6.21; N, 17.27. Found: C, 66.81; H, 6.37; N, 17.45.

***N*-(4-(Hydrazinecarbonyl)phenyl)-2-(4-phenylpiperazin-1-yl)acetamide** (**3 h**) Yield 80%; buff solid; m.p. 260 °C; IR (KBr): 3326, 3268 (NH_2_, NH), 1665, 1627 (C = O); ^1^H-NMR (400 MHz, DMSO-*d*_*6*_) *δ* 2.67 (brs, 4 H, (CH_2_)_2_), 3.22 (brs, 6 H, CH_2_-C = O, (CH_2_)_2_), 4.46 (brs, 2 H, NH_2_), 6.76 (t, 1H, Ar-H, *J* = 7.2 Hz), 6.93 (d, 2 H, Ar-H, *J* = 8.4 Hz), 7.19 (t, 2 H, Ar-H, *J* = 7.6 Hz), 7.71 (d, 2 H, Ar-H, *J* = 8.4 Hz), 7.79 (d, 2 H, Ar-H, *J* = 8.4 Hz), 9.66 (s, 1H, NH), 9.97 (s, 1H, NH); ^13^C-NMR (125 MHz, DMSO-*d*_*6*_) *δ* 48.2, 52.9, 62.0, 115.8, 119.1, 119.2, 128.1, 128.4, 129.3, 141.4, 151.2, 165.9, 169.1; Ms m/z (EI) 354.18 (M^+^+1) (14.22%); Anal. Calcd for C_19_H_23_N_5_O_2_: C, 64.57; H, 6.56; N, 19.82. Found: C, 64.39; H, 6.70; N, 20.04.

***N*-(4-(Hydrazinecarbonyl)phenyl)-2-(4-(phenylsulfonyl)piperazin-1-yl)acetamide** (**6a**) Yield 60%; white solid; m.p. 228 °C; IR (KBr): 3330, 3290 (NH_2_, NH), 1685, 1654 (C = O); ^1^H-NMR (400 MHz, DMSO-*d*_*6*_) *δ* 2.59 (brs, 4 H, (CH_2_)_2_), 2.98 (brs, 4 H, (CH_2_)_2_), 3.16 (s, 2 H, CH_2_-C = O), 4.42 (brs, 2 H, NH_2_), 7.63–7.69 (m, 4 H, Ar-H), 7.73–7.77 (m, 5 H, Ar-H), 9.64 (s, 1H, NH), 9.84 (s, 1H, NH); ^13^C-NMR (100 MHz, DMSO-*d*_*6*_) *δ* 46.1, 52.2, 61.7, 119.6, 128.03, 128.07, 128.4, 129.9, 133.7, 135.4, 141.4, 166.9, 168.8; Anal. Calcd for C_19_H_23_N_5_O_4_S: C, 54.66; H, 5.55; N, 16.78. Found: C, 54.80; H, 5.69; N, 17.06.

**2-(4-((4-Chlorophenyl)sulfonyl)piperazin-1-yl)-*N*-(4-(hydrazinecarbonyl)phenyl)acetamide** (**6b**) Yield 57%; white solid; m.p. 215 °C; IR (KBr): 3325, 3260 (NH_2_, NH), 1670, 1626 (C = O); ^1^H-NMR (400 MHz, DMSO-*d*_*6*_) *δ* 2.60 (brs, 4 H, (CH_2_)_2_), 3.00 (brs, 4 H, (CH_2_)_2_), 3.17 (s, 2 H, CH_2_-C = O), 4.43 (brs, 2 H, NH_2_), 7.64 (d, 2 H, Ar-H, *J* = 8.8 Hz), 7.74–7.79 (m, 6 H, Ar-H), 9.64 (s, 1H, NH), 9.81 (s, 1H, NH); ^13^C-NMR (100 MHz, DMSO-*d*_*6*_) *δ* 46.0, 52.2, 61.3, 119.2, 128.0, 128.4, 129.9, 130.0, 134.4, 138.7, 141.4, 165.9, 168.7; Anal. Calcd for C_19_H_22_ClN_5_O_4_S: C, 50.50; H, 4.91; N, 15.50. Found: C, 50.78; H, 5.07; N, 15.39.

**2-(4-((4-Bromophenyl)sulfonyl)piperazin-1-yl)-*N*-(4-(hydrazinecarbonyl)phenyl)acetamide** (**6c**) Yield 63%; white solid; m.p. 252 °C; IR (KBr): 3340, 3298 (NH_2_, NH), 1670, 1643 (C = O); ^1^H-NMR (400 MHz, DMSO-*d*_*6*_) *δ* 2.60 (brs, 4 H, (CH_2_)_2_), 3.00 (brs, 4 H, (CH_2_)_2_), 3.17 (s, 2 H, CH_2_-C = O), 4.43 (brs, 2 H, NH_2_), 7.64–7.70 (m, 4 H, Ar-H), 7.75 (d, 2 H, Ar-H, *J* = 8.4 Hz), 7.88 (d, 2 H, Ar-H, *J* = 8.8 Hz), 9.64 (s, 1H, NH), 9.81 (s, 1H, NH); ^13^C-NMR (100 MHz, DMSO-*d*_*6*_) *δ* 46.1, 52.0, 61.3, 119.2, 127.7, 128.0, 128.4, 130.0, 133.0, 134.8, 141.3, 166.0, 168.8; Anal. Calcd for C_19_H_22_ClN_5_O_4_S: C, 45.97; H, 4.47; N, 14.11. Found: C, 46.19; H, 4.56; N, 14.34.

***N*-(4-(Hydrazinecarbonyl)phenyl)-2-(4-tosylpiperazin-1-yl)acetamide** (**6d**) Yield 60%; white solid; m.p. 170 °C; IR (KBr): 3340, 3228 (NH_2_, NH), 1670, 1647 (C = O); ^1^H-NMR (400 MHz, DMSO-*d*_*6*_) *δ* 2.42 (s, 3 H, CH_3_), 2.58 (brs, 4 H, (CH_2_)_2_), 2.95 (brs, 4 H, (CH_2_)_2_), 3.16 (s, 2 H, CH_2_-C = O), 4.43 (brs, 2 H, NH_2_), 7.46 (d, 2 H, Ar-H, *J* = 8.4 Hz), 7.63 (d, 4 H, Ar-H, *J* = 8.4 Hz), 7.74 (d, 2 H, Ar-H, *J* = 8.8 Hz), 9.64 (s, 1H, NH), 9.82 (s, 1H, NH); ^13^C-NMR (100 MHz, DMSO-*d*_*6*_) *δ* 21.2, 46.0, 52.2, 61.7, 119.4, 128.0, 128.1, 128.4, 130.3, 132.4, 141.4, 143.9, 165.9, 168.7; Anal. Calcd for C_20_H_25_N_5_O_4_S: C, 55.67; H, 5.84; N, 16.23. Found: C, 55.94; H, 6.01; N, 16.59.

***N*-(4-(Hydrazinecarbonyl)phenyl)-2-(4-((4-methoxyphenyl)sulfonyl)piperazin-1-yl)acetamide** (**6e**) Yield 69%; white solid; m.p. 193 °C; IR (KBr): 3336, 3248 (NH_2_, NH), 1674, 1647 (C = O); ^1^H-NMR (400 MHz, DMSO-*d*_*6*_) *δ* 2.59 (brs, 4 H, (CH_2_)_2_), 2.95 (brs, 4 H, (CH_2_)_2_), 3.16 (s, 2 H, CH_2_-C = O), 3.87 (s, 3 H, OCH_3_), 4.42 (brs, 2 H, NH_2_), 7.16 (d, 2 H, Ar-H, *J* = 8 Hz), 7.63–7.70 (m, 4 H, Ar-H), 7.75 (d, 2 H, Ar-H, *J* = 8 Hz), 9.62 (s, 1H, NH), 9.80 (s, 1H, NH); ^13^C-NMR (100 MHz, DMSO-*d*_*6*_) *δ* 45.6, 51.6, 55.5, 60.8, 114.5, 118.9, 126.3, 127.5, 127.9, 129.8, 140.9, 162.8, 165.1, 168.1; Anal. Calcd for C_20_H_25_N_5_O_5_S: C, 53.68; H, 5.63; N, 15.65. Found: C, 53.83; H, 5.78; N, 15.93.

***N*-(4-(Hydrazinecarbonyl)phenyl)-2-(4-((4-(trifluoromethyl)phenyl)sulfonyl)piperazin-1-yl)acetamide** (**6f**) Yield 68%; white solid; m.p. 220 °C; IR (KBr): 3344, 3248 (NH_2_, NH), 1674, 1647 (C = O); ^1^H-NMR (400 MHz, DMSO-*d*_*6*_) *δ* 2.61 (brs, 4 H, (CH_2_)_2_), 3.05 (brs, 4 H, (CH_2_)_2_), 3.18 (s, 2 H, CH_2_-C = O), 4.43 (brs, 2 H, NH_2_), 7.63 (d, 2 H, Ar-H, *J* = 8 Hz), 7.75 (d, 2 H, Ar-H, *J* = 8.4 Hz), 7.97 (d, 2 H, Ar-H, *J* = 8 Hz), 8.04 (d, 2 H, Ar-H, *J* = 8.4 Hz), 9.63 (s, 1H, NH), 9.80 (s, 1H, NH); ^13^C-NMR (100 MHz, DMSO-*d*_*6*_) *δ* 45.6, 51.6, 61.0, 118.9, 126.6 (d, *J* = 4 Hz), 127.6, 128.0, 128.5, 132.6, 133.1, 139.1, 140.9, 165.4, 168.3; Anal. Calcd for C_20_H_22_F_3_N_5_O_4_S: C, 49.48; H, 4.57; N, 14.43. Found: C, 49.70; H, 4.71; N, 14.36.

**General procedure for the synthesis of** ***N*-(1**,**3-dioxoisoindolin-2-yl)-4-(2-(substituted)acetamido)benzamide 4a-h and 7a-f**.

A mixture of acid hydrazide **3a-h** or **6a-f** (0.002 mol), phthalic anhydride (0.3 gm, 0.002 mol) and anhydrous sodium acetate (0.41 gm, 0.005 mol) in 15 ml glacial acetic acid was refluxed for 4 h. After cooling to room temperature the mixture was poured into ice-cold water and the solid was filtered off followed by re-crystallization from absolute ethanol to get the final compounds **4a-h** and **7a-f**.

**4-(2-(Dimethylamino)acetamido)-*N*-(1**,**3-dioxoisoindolin-2-yl)benzamide** (**4a**) Yield 55%; white solid; m.p. 200 °C; IR (KBr, cm^− 1^): 3252 (NH), 1739 (C = O), 1675 (C = O); ^1^H-NMR (400 MHz, DMSO-*d*_*6*_) *δ* 2.29 (s, 6 H, N-(CH_3_)_2_), 3.13 (s, 2 H, CH_2_-C = O), 7.85 (d, 2 H, Ar-H, *J* = 8.8 Hz), 7.93–8.02 (m, 6 H, Ar-H), 10.08 (s, 1H, NH), 11.20 (s, 1H, NH); ^13^C-NMR (100 MHz, DMSO-*d*_*6*_) *δ* 45.6, 63.7, 119.3, 124.3, 125.4, 129.2, 129.9, 135.8, 143.1, 165.2, 165.9, 169.8; HRMS (ESI) calculated for C_19_H_18_N_4_O_4_ [M + H]^+^: 367.14064, found 367.14240.

**4-(2-(Diethylamino)acetamido)-*N*-(1**,**3-dioxoisoindolin-2-yl)benzamide** (**4b**) Yield 71%; white solid; m.p. 205 °C; IR (KBr, cm^− 1^): 3228 (NH), 1739 (C = O), 1670 (C = O); ^1^H-NMR (400 MHz, DMSO-*d*_*6*_) *δ* 1.00 (t, 6 H, (CH_3_)_2_, *J* = 7.2 Hz), 2.58 (q, 4 H, (CH_2_)_2_, *J* = 7.2 Hz) 3.21 (s, 2 H, CH_2_-C = O), 7.85 (d, 2 H, Ar-H, *J* = 8.4 Hz), 7.95–8.02 (m, 6 H, Ar-H), 9.99 (s, 1H, NH), 11.19 (s, 1H, NH); ^13^C-NMR (100 MHz, DMSO-*d*_*6*_) *δ* 12.1, 47.8, 58.0, 119.2, 124.3, 125.6, 129.2, 130.0, 135.7, 142.8, 165.2, 165.9, 171.0; HRMS (ESI) calculated for C_21_H_22_N_4_O_4_ [M + H]^+^: 395.17194, found 395.17421.

***N*-(1**,**3-Dioxoisoindolin-2-yl)-4-(2-(dipropylamino)acetamido)benzamide** (**4c**) Yield 79%; white solid; m.p. 172 °C; HPLC: purity 99.41%, R_t_= 6.68 min; IR (KBr, cm^− 1^): 3250 (NH), 1739 (C = O), 1674 (C = O); ^1^H-NMR (400 MHz, DMSO-*d*_*6*_) *δ* 0.86 (t, 6 H, *J* = 7.2 Hz, (CH_3_)_2_), 1.42–1.51 (m, 4 H, (*CH*_*2*_-CH_3_)_2_), 2.49–2.53 (m, 4 H, (*CH*_*2*_-CH_2_-CH_3_)_2_), 3.24 (s, 2 H, CH_2_-C = O), 7.82 (d, 2 H, Ar-H, *J* = 8.4 Hz), 7.95–8.03 (m, 6 H, Ar-H), 9.91 (s, 1H, NH), 11.20 (s, 1H, NH); ^13^C-NMR (100 MHz, DMSO-*d*_*6*_) *δ* 12.2, 20.1, 56.5, 58.7, 119.0, 124.2, 125.5, 129.3, 129.9, 135.8, 142.6, 165.2, 165.8, 170.8; HRMS (ESI) calculated for C_23_H_26_N_4_O_4_ [M + H]^+^: 423.20324, found 423.20613.

**4-(2-(Diisopropylamino)acetamido)-*N*-(1**,**3-dioxoisoindolin-2-yl)benzamide** (**4d**) Yield 81%; white solid; m.p. 240 °C; IR (KBr, cm^− 1^): 3259 (NH), 1735 (C = O), 1689 (C = O), 1678 (C = O); ^1^H-NMR (400 MHz, DMSO-*d*_*6*_) *δ* 1.03 (d, 12 H, *J* = 6.4 Hz, (CH(*CH*_*3*_)_2_)_2_), 3.03–3.07 (m, 2 H, (*CH*(CH_3_)_2_)_2_), 3.16 (s, 2 H, CH_2_-C = O), 7.85 (d, 2 H, Ar-H, *J* = 8.8 Hz), 7.95–8.02 (m, 6 H, Ar-H), 9.86 (s, 1H, NH), 11.22 (s, 1H, NH); ^13^C-NMR (100 MHz, DMSO-*d*_*6*_) *δ* 20.5, 50.2, 50.3, 119.1, 124.3, 125.7, 129.3, 129.8, 135.8, 142.4, 165.2, 165.9, 172.6; HRMS (ESI) calculated for C_23_H_26_N_4_O_4_ [M + H]^+^: 423.20324, found 423.20473.

***N*-(1**,**3-Dioxoisoindolin-2-yl)-4-(2-(piperidin-1-yl)acetamido)benzamide** (**4e**) Yield 74%; white solid; m.p. 250 °C; IR (KBr, cm^− 1^): 3263 (NH), 1743 (C = O), 1678 (C = O); ^1^H-NMR (400 MHz, DMSO-*d*_*6*_) *δ* 1.35–1.37 (m, 2 H, CH_2_), 1.51–1.54 (m, 4 H, (CH_2_)_2_), 2.42–2.47 (m, 4 H, (CH_2_)_2_), 3.08 (s, 2 H, CH_2_-C = O), 7.80 (d, 2 H, Ar-H, *J* = 7.6 Hz), 7.91–7.98 (m, 6 H, Ar-H), 9.97 (s, 1H, NH), 11.16 (s, 1H, NH); ^13^C-NMR (100 MHz, DMSO-*d*_*6*_) *δ* 24.2, 26.0, 54.1, 63.2, 119.4, 124.2, 125.6, 129.2, 130.0, 135.9, 142.9, 165.2, 165.9, 169.8; HRMS (ESI) calculated for C_22_H_22_N_4_O_4_ [M + H]^+^: 407.17194, found 407.17366.

***N*-(1**,**3-Dioxoisoindolin-2-yl)-4-(2-morpholinoacetamido)benzamide** (**4f**) Yield 70%; white solid; m.p. 255 °C; IR (KBr, cm^− 1^): 3278 (NH), 1739 (C = O), 1681 (C = O); ^1^H-NMR (400 MHz, DMSO-*d*_*6*_) *δ* 2.53 (brs, 4 H, (CH_2_)_2_), 3.19 (s, 2 H, CH_2_-C = O), 3.64 (brs, 4 H, (CH_2_)_2_), 7.83 (d, 2 H, Ar-H, *J* = 8.8 Hz), 7.94–8.02 (m, 6 H, Ar-H), 10.10 (s, 1H, NH), 11.14 (s, 1H, NH); ^13^C-NMR (100 MHz, DMSO-*d*_*6*_) *δ* 53.6, 62.4, 66.5, 119.2, 124.3, 125.6, 129.2, 129.9, 135.8, 143.0, 165.2, 165.9, 169.3; HRMS (ESI) calculated for C_21_H_20_N_4_O_5_ [M + H]^+^: 409.15120, found 409.15371.

**4-(2-(3**,**4-Dihydroisoquinolin-2(1*H*)-yl)acetamido)-*N*-(1**,**3-dioxoisoindolin-2-yl)benzamide** (**4 g**) Yield 72%; white solid; m.p. 200 °C; IR (KBr, cm^− 1^): 3240 (NH), 1743 (C = O), 1685 (C = O); ^1^H-NMR (400 MHz, DMSO-*d*_*6*_) *δ* 2.84–2.90 (m, 4 H, CH_2_-CH_2_-N), 3.38 (s, 2 H, CH_2_-C = O), 3.75 (s, 2 H, CH_2_-N), 7.06–7.13 (m, 4 H, Ar-H), 7.86 (d, 2 H, Ar-H, *J* = 8.8 Hz), 7.95–8.02 (m, 6 H, Ar-H), 10.15 (s, 1H, NH), 11.21 (s, 1H, NH); ^13^C-NMR (100 MHz, DMSO-*d*_*6*_) *δ* 28.9, 50.8, 55.5, 62.0, 119.4, 124.3, 125.6, 125.9, 126.5, 126.8, 128.9, 129.2, 129.9, 134.2, 134.8, 135.8, 143.1, 165.2, 165.9, 169.6; HRMS (ESI) calculated for C_26_H_22_N_4_O_4_ [M + H]^+^: 455.17194, found 455.17561.

***N*-(1**,**3-Dioxoisoindolin-2-yl)-4-(2-(4-phenylpiperazin-1-yl)acetamido)benzamide** (**4 h**) Yield 60%; white solid; m.p. 243 °C; IR (KBr, cm^− 1^): 3270 (NH), 1735 (C = O), 1685 (C = O); ^1^H-NMR (400 MHz, DMSO-*d*_*6*_) *δ* 2.70 (brs, 4 H, (CH_2_)_2_), 3.22 (d, 6 H, CH_2_-C = O, (CH_2_)_2_, *J* = 16.8 Hz), 6.76 (t, 1H, Ar-H, *J* = 7.2 Hz), 6.94 (d, 2 H, Ar-H, *J* = 8 Hz), 7.20 (t, 2 H, Ar-H, *J* = 7.6 Hz), 7.85 (d, 2 H, Ar-H, *J* = 8.4 Hz), 7.95 (m, 6 H, Ar-H), 10.13 (s, 1H, NH), 11.21 (s, 1H, NH); ^13^C-NMR (125 MHz, DMSO-*d*_*6*_) *δ* 48.5, 53.1, 62.0, 115.8, 119.2, 119.3, 124.3, 125.6, 129.2, 129.3, 129.8, 135.8, 143.0, 151.4, 165.2, 165.9, 169.3; HRMS (ESI) calculated for C_27_H_25_N_5_O_4_ [M + H]^+^: 484.19848, found 484.20082.

***N*-(1**,**3-Dioxoisoindolin-2-yl)-4-(2-(4-(phenylsulfonyl)piperazin-1-yl)acetamido)benzamide (7a)** Yield 75%; buff solid; m.p. 256 °C; HPLC: purity 99.6%, R_t_= 6.25 min; IR (KBr, cm^− 1^): 3278 (NH), 1739 (C = O), 1685 (C = O); ^1^H-NMR (400 MHz, DMSO-*d*_*6*_) *δ* 2.61 (brs, 4 H, (CH_2_)_2_), 2.99 (brs, 4 H, (CH_2_)_2_), 3.20 (s, 2 H, CH_2_-C = O), 7.67–7.70 (m, 2 H, Ar-H), 7.74–7.78 (m, 5 H, Ar-H), 7.90 (d, 2 H, Ar-H, *J* = 8.8 Hz), 7.96–8.02 (m, 4 H, Ar-H), 9.99 (s, 1H, NH), 11.18 (s, 1H, NH); ^13^C-NMR (100 MHz, DMSO-*d*_*6*_) *δ* 46.1, 52.1, 61.6, 119.5, 124.3, 125.6, 128.0, 129.1, 129.93, 129.94, 133.7, 135.4, 135.8, 142.9, 165.2, 165.9, 169.1; HRMS (ESI) calculated for C_27_H_25_N_5_O_6_S [M + H]^+^: 548.16038, found 548.16380.

**4-(2-(4-((4-Chlorophenyl)sulfonyl)piperazin-1-yl)acetamido)-*N*-(1**,**3-dioxoisoindolin-2-yl)benzamide** (**7b**) Yield 71%; buff solid; m.p. 270 °C; IR (KBr, cm^− 1^): 3248 (NH), 1739 (C = O), 1670 (C = O); ^1^H-NMR (400 MHz, DMSO-*d*_*6*_) *δ* 2.67 (brs, 4 H, (CH_2_)_2_), 3.06 (brs, 4 H, (CH_2_)_2_), 3.26 (s, 2 H, CH_2_-C = O), 7.79–7.85 (m, 6 H, Ar-H), 7.96 (d, 2 H, Ar-H, *J* = 8.4 Hz), 8.00-8.07 (m, 4 H, Ar-H), 10.02 (s, 1H, NH), 11.23 (s, 1H, NH); ^13^C-NMR (100 MHz, DMSO-*d*_*6*_) *δ* 46.1, 52.0, 61.5, 119.5, 124.3, 125.6, 129.1, 129.96, 129.98, 130.1, 134.5, 135.7, 138.7, 143.0, 165.2, 165.9, 169.1; HRMS (ESI) calculated for C_27_H_24_ClN_5_O_6_S [M + H]^+^: 582.121410, found 582.12278.

**4-(2-(4-((4-Bromophenyl)sulfonyl)piperazin-1-yl)acetamido)-*N*-(1**,**3-dioxoisoindolin-2-yl)benzamide** (**7c**) Yield 76%; white solid; m.p. 256 °C; IR (KBr, cm^− 1^): 3263 (NH), 1739 (C = O), 1681 (C = O); ^1^H-NMR (400 MHz, DMSO-*d*_*6*_) *δ* 2.62 (brs, 4 H, (CH_2_)_2_), 3.02 (brs, 4 H, (CH_2_)_2_), 3.21 (s, 2 H, CH_2_-C = O), 7.68 (d, 2 H, Ar-H, *J* = 9 Hz), 7.78 (d, 2 H, Ar-H, *J* = 9 Hz), 8.88–7.99 (m, 8 H, Ar-H), 9.96 (s, 1H, NH), 11.18 (s, 1H, NH); ^13^C-NMR (100 MHz, DMSO-*d*_*6*_) *δ* 45.6, 51.6, 61.0, 119.0, 123.8, 125.1, 127.3, 128.6, 129.4, 129.5, 132.5, 134.3, 135.3, 142.4, 164.7, 165.4, 168.6; HRMS (ESI) calculated for C_27_H_24_BrN_5_O_6_S [M + H]^+^: 626.07090, found 626.0728.

***N*-(1**,**3-Dioxoisoindolin-2-yl)-4-(2-(4-tosylpiperazin-1-yl)acetamido)benzamide** (**7d**) Yield 68%; buff solid; m.p. 230 °C; IR (KBr, cm^− 1^): 3265 (NH), 1739 (C = O), 1685 (C = O); ^1^H-NMR (400 MHz, DMSO-*d*_*6*_) *δ* 2.48 (s, 3 H, CH_3_), 2.66 (brs, 4 H, (CH_2_)_2_), 3.02 (brs, 4 H, (CH_2_)_2_), 3.25 (s, 2 H, CH_2_-C = O), 7.52 (d, 2 H, Ar-H, *J* = 8 Hz), 7.69 (d, 2 H, Ar-H, *J* = 8.4 Hz), 7.82 (d, 2 H, Ar-H, *J* = 8.8 Hz), 7.96–8.07 (m, 6 H, Ar-H), 10.02 (s, 1H, NH), 11.23 (s, 1H, NH); ^13^C-NMR (100 MHz, DMSO-*d*_*6*_) *δ* 21.2, 46.0, 52.2, 61.3, 119.5, 124.3, 125.5, 128.1, 129.1, 129.8, 130.3, 132.4, 135.8, 142.9, 144.1, 165.2, 165.9, 169.1; HRMS (ESI) calculated for C_28_H_27_N_5_O_6_S [M + H]^+^: 562.17603, found 562.18081.

***N*-(1**,**3-Dioxoisoindolin-2-yl)-4-(2-(4-((4-methoxyphenyl)sulfonyl)piperazin-1-yl)acetamido)benzamide** (**7e**) Yield 71%; white solid; m.p. 303 °C; IR (KBr, cm^− 1^): 3236 (NH), 1739 (C = O), 1678 (C = O); ^1^H-NMR (400 MHz, DMSO-*d*_*6*_) *δ* 2.61 (brs, 4 H, (CH_2_)_2_), 2.96 (brs, 4 H, (CH_2_)_2_), 3.20 (s, 2 H, CH_2_-C = O), 3.87 (s, 3 H, OCH_3_), 7.16 (d, 2 H, Ar-H, *J* = 9 Hz), 7.68 (d, 2 H, Ar-H, *J* = 9 Hz), 7.77 (d, 2 H, Ar-H, *J* = 9 Hz), 7.92–8.02 (m, 6 H, Ar-H), 9.97 (s, 1H, NH), 11.17 (s, 1H, NH); ^13^C-NMR (100 MHz, DMSO-*d*_*6*_) *δ* 45.7, 51.6, 55.7, 61.1, 114.5, 119.0, 123.8, 125.1, 126.4, 128.7, 129.4, 129.8, 135.3, 142.5, 162.8, 164.7, 165.4, 168.6; HRMS (ESI) calculated for C_28_H_27_N_5_O_7_S [M + H]^+^: 578.17095, found 578.17104.

***N*-(1**,**3-Dioxoisoindolin-2-yl)-4-(2-(4-((4-(trifluoromethyl)phenyl)sulfonyl)piperazin-1-yl)acetamido)benzamide** (**7f**) Yield 70%; white solid; m.p. 278 °C; IR (KBr, cm^− 1^): 3259 (NH), 1743 (C = O), 1674 (C = O); ^1^H-NMR (400 MHz, DMSO-*d*_*6*_) *δ* 2.63 (brs, 4 H, (CH_2_)_2_), 3.07 (brs, 4 H, (CH_2_)_2_), 3.22 (s, 2 H, CH_2_-C = O), 7.76 (d, 2 H, Ar-H, *J* = 8.8 Hz), 7.91–8.01 (m, 8 H, Ar-H), 8.05 (d, 2 H, Ar-H, *J* = 8.8 Hz), 9.96 (s, 1H, NH), 11.16 (s, 1H, NH); ^13^C-NMR (100 MHz, DMSO-*d*_*6*_) *δ* 45.6, 51.5, 60.9, 119.0, 120.4, 123.8, 125.1, 126.6 (d, *J* = 5 Hz), 128.5, 128.6, 129.4, 133.0, 135.3, 139.2, 142.4, 164.7, 165.4, 168.6; HRMS (ESI) calculated for C_28_H_24_F_3_N_5_O_6_S [M + H]^+^: 616.14777, found 616.15011.

### Biological evaluation

In vitro **determination of COX-1 and COX-2 inhibitory activity**.

The capability of all the target compounds, **4a-h** and **7a-f** as well as the reference drugs, indomethacin, celecoxib and meloxicam, to inhibit both COX-1 and COX-2 isoenzymes was carried out by using fluorometric COX-1 and COX-2 inhibitor screening kits supplied by Biovision Incorporated, CA, USA (catalog number K548-100 and K547-100, respectively) according to procedures supplied by the manufacturer.

In vitro **determination of 5-LOX inhibitory activity**.

The ability of the target compounds to inhibit 5-LOX was determined by using the 5-LOX inhibition assay kit obtained from Biovision Incorporated, CA, USA (catalog number K980-100, following the manufacturer’s instructions.

### Cell viability

Cell viability was performed by using MTT assay according to reported procedure [68, 69].

#### NO inhibtion assay

The level of nitrite, a key for NO production, was determined following the Griess reaction according to the previously reported method [69].

### Determination of PGE-2, IL-6 and α-TNF expression

The effect of compounds **4c** and **7a** on the expression of PGE-2, IL-6 and α-TNF in LPS-stimulated macrophages at 10 µM was determined *via* quantitative reverse transcription polymerase chain reaction (qRT-PCR). The analysis was performed by using the BIORAD iScript™ One-Step RT-PCR Kit with SYBR^®^ Green, following the manufacturer’s instructions.

#### Molecular Docking

Molecular docking studies were performed by Drug Discovery 2021 Client. COX-2 (PDB: 5KIR) and 5-LOX (PDB: 6N2W) enzymes were downloaded from the protein data bank. The protein preparation for docking was carried by deleting water and adding hydrogen atoms to the structure with their standard geometry. The binding site was determined *via* the co-crystallized ligands. Energy minimizations were performed. Molecular docking studies were done by using CDOCKER protocol. First the validation step of the docking protocol was carried out by re-docking of the co-crystallized ligand into the active site of the enzymes. The validated docking protocol was applied to perform the docking study of compounds **4c** and **7a**.

#### Molecular dynamic simulations

To investigate the structural stability and binding affinity of the enzyme COX-2–compound **4c**, COX-2–compound **7a**, and 5-LOX-compound **4c** complexes, a conventional all-atom molecular dynamics (MD) simulation was carried out. A 200 ns simulation trajectory was generated using the GROMACS 2024 software package for each system [70]. Initial system setup for the complexes was performed *via* the CHARMM-GUI solution builder interface [71–74], with COX-2 system enclosed in a 9.6 nm and 5LOX enclosed in a 12 nm cubic simulation box solvated with TIP3P water molecules, ensuring a minimum solute-solvent distance of 1 nm. To neutralize the system and mimic physiological conditions, Na⁺ and Cl⁻ ions were added to achieve a final salt concentration of 0.154 M. The protein, solvent, and ions were modelled using the CHARMM36m force field, whereas the compounds were parameterized using the CHARMM General Force Field (CGenFF).

Molecular dynamics (MD) calculations were executed under three-dimensional periodic boundary conditions. Before the production run, the system was energetically relaxed by steepest-descent minimization until the largest atomic force fell below 100 kJ mol⁻¹ nm⁻¹ or until 100,000 steps had elapsed. Subsequent equilibration was performed in two stages: at first an NVT phase at 310 K was initiated and the temperature was maintained by the velocity-rescale thermostat. After that, an NPT phase at 1 atm was initiated, and the pressure was maintained with the Berendsen barostat while temperature was again controlled by the same thermostat [75]. Production simulation was conducted in the NPT ensemble with the Nosé–Hoover thermostat (310 K) and the Parrinello–Rahman barostat (1 atm) [76]. Covalent bonds involving hydrogen were constrained with the LINCS algorithm [77], permitting a 2 fs integration timestep during production while 1 fs was employed during equilibration. Long-range electrostatics were evaluated using the particle-mesh Ewald (PME) method with a cutoff of 1.2 nm [78]. Newtonian equations of motion were integrated *via* the leap-frog algorithm. Trajectory frames were saved every 0.1 ns, yielding 2,000 snapshots. After removal of periodicity artifacts and verification of structural integrity, trajectories were processed with VMD TK scripting [79]. Structural descriptors comprised backbone-root-mean-square deviation (RMSD) of the protein, heavy-atom RMSD of ligand **4c** or **7a** Cα-root-mean-square fluctuation (RMSF), radius of gyration (Rg), solvent-accessible surface area (SASA), intermolecular hydrogen-bond formation, and center-of-mass (COM) distance between ligand and receptor. Residue-resolved interaction profiles were generated with the Python package ProLIF [80], enabling quantitative classification and temporal mapping of non-covalent contacts between the test compound and each enzyme throughout the simulations.

#### Binding free energy calculation using MM-GBSA

The binding free energies (ΔG) of ligand **4c** or **7a** with COX-2 protein and **4c** with 5-LOX were estimated by the Molecular Mechanics/Generalized Born Surface Area (MM-GBSA) protocol implemented in the gmx_MMPBSA package [81, 82]. The method combines gas-phase molecular mechanics energies with an implicit GB solvation term to yield ΔG. To quantify residue-specific energetic contributions, per-residue decomposition was performed for all amino acids whose side-chain heavy atoms lay within 1.0 nm of the ligand. All computations were carried out at physiological ionic strength (0.154 M NaCl). The Generalized Born model was invoked with the igb = 5 parameter set, while internal and external dielectric constants were fixed at 1.0, 78.5, respectively. The governing thermodynamic cycle for ΔG is given by Eq. 1:

1 $$ \Delta G = \left\langle {Gcomplex - (Greceptor\: + \:Gligand)} \right\rangle $$

Here, < > denotes the ensemble average of the free energies for the complex, receptor, and ligand calculated across the analyzed simulation frames. In this study, the entire 200 ns trajectory was utilized, with snapshots sampled every 10 frames, resulting in a total of 200 frames for the energy calculations. The various energy components can be determined according to Eq. 2 through 6:

2 $$ \Delta Gbinding\: = \Delta H - T\Delta S $$

3 $$ \Delta H = \Delta Egas + \Delta Esol $$

4 $$ \Delta Egas = \Delta Eele + \Delta EvdW $$

5 * Δ Esolv = EGB + ESA *

6 * ESA = γ .SASA *

where:

ΔH represents the enthalpy, which is derived from the gas-phase energy (E_gas_​) and the solvation-free energy (E_sol_​). The term − TΔS accounts for the entropic contribution to the binding free energy. The gas-phase energy (E_gas_​) comprises electrostatic (E_ele_​) and van der Waals (E_vdw_​) energy terms. The solvation-free energy (E_sol_​) is calculated as the sum of the polar solvation energy (E_GB_​) and the non-polar solvation energy (E_SA_​), with the latter estimated based on the solvent-accessible surface area [83, 84].

## Supplementary Information

Below is the link to the electronic supplementary material.

Supplementary Material 1

## Data Availability

No datasets were generated or analysed during the current study.
