# Supplementary material for: Design, synthesis, biological evaluation and computational studies of novel phthalimides as dual COX-2/5-LOX inhibitors
Source: Mol Divers. 2026 Jan 21;30(5):7155–83. doi: 10.1007/s11030-025-11431-z (PMC13635043; doi:10.1007/s11030-025-11431-z)
Supplement: Supplementary file 1 [file 11030_2025_11431_MOESM1_ESM.docx]

**Supporting information**

**Design, synthesis, biological evaluation and computational studies of novel phthalimides as dual COX-2/5-LOX inhibitors**

**Rasha M. Hassan^1*^, Mohammed S. Abdel-Maksoud^1^, May A. El-Manawaty^2^, Ibrahim M. Ibrahim^3^, Walaa H. Abd-Allah^4^, Aida A. El-Azzouny^1^, Mohamed N. Aboul-Enein^1*^**

^1^ Medicinal and Pharmaceutical Chemistry Department, Pharmaceutical and Drug Industries Research Institute, National Research Centre (ID: 60014618), P.O. 12622, Dokki, Giza, Egypt.

^2^ Pharmacognosy Department, National Research Centre (ID: 60014618), Dokki, Giza 12622, Egypt.

^3^ Biophysics Department, Faculty of Science, Cairo University, Giza 12613, Egypt.

^4^ Pharmaceutical Chemistry Department, Collage of Pharmaceutical Science and Drug Manufacturing, Misr University for Science and Technology, P.O. 77, 6th of October City, Giza, Egypt.

*Corresponding author: Rasha M. Hassan, Email: [rashahassan_pharma@yahoo.ca](mailto:rashahassan_pharma@yahoo.ca), Mohamed N. Aboul-Enein, Email: [mnaboulenein@yahoo.com](mailto:mnaboulenein@yahoo.com)

**Spectral data of the synthesized compounds**

**Figure S.1**: ^1^H NMR spectrum of compound **2a**.

**Figure S.2**: ^13^C NMR spectrum of compound **2a**.

**Figure S.3**: ^1^H NMR spectrum of compound **2b**.

**Figure S.4**: ^13^C NMR spectrum of compound **2b**.

**Figure S.5**: ^1^H NMR spectrum of compound **2c**.

**Figure S.6**: ^13^C NMR spectrum of compound **2c**.

**Figure S.7**: ^1^H NMR spectrum of compound **2d**.

**Figure S.8**: ^13^C NMR spectrum of compound **2d**.

**Figure S.9**: ^1^H NMR spectrum of compound **2e**.

**Figure S.10**: ^13^C NMR spectrum of compound **2e**.

**Figure S.11**: ^1^H NMR spectrum of compound **2f**.

**Figure S.12**: ^13^C NMR spectrum of compound **2f**.

**Figure S.13**: ^1^H NMR spectrum of compound **2g**.

**Figure S.14**: ^13^C NMR spectrum of compound **2g**.

**Figure S.15**: ^1^H NMR spectrum of compound **2h**.

**Figure S.16**: ^13^C NMR spectrum of compound **2h**.

**Figure S.17**: ^1^H NMR spectrum of compound **5a**.

**Figure S.18**: ^13^C NMR spectrum of compound **5a**.

**Figure S.19**: ^1^H NMR spectrum of compound **5b**.

**Figure S.20**: ^13^C NMR spectrum of compound **5b**.

**Figure S.21**: ^1^H NMR spectrum of compound **5c**.

**Figure S.22**: ^13^C NMR spectrum of compound **5c**.

**Figure S.23**: ^1^H NMR spectrum of compound **5d**.

**Figure S.24**: ^13^C NMR spectrum of compound **5d**.

**Figure S.25**: ^1^H NMR spectrum of compound **5e**.

**Figure S.26**: ^13^C NMR spectrum of compound **5e**.

**Figure S.27**: ^1^H NMR spectrum of compound **5f**.

**Figure S.28**: ^13^C NMR spectrum of compound **5f**.

**Figure S.29**: ^1^H NMR spectrum of compound **3a**.

**Figure S.30**: ^13^C NMR spectrum of compound **3a**.

**Figure S.31**: ^1^H NMR spectrum of compound **3b**.

**Figure S.32**: ^13^C NMR spectrum of compound **3b**.

**Figure S.33**: ^1^H NMR spectrum of compound **3c**.

**Figure S.34**: ^13^C NMR spectrum of compound **3c**.

**Figure S.35**: ^1^H NMR spectrum of compound **3d**.

**Figure S.36**: ^13^C NMR spectrum of compound **3d**.

**Figure S.37**: ^1^H NMR spectrum of compound **3e**.

**Figure S.38**: ^13^C NMR spectrum of compound **3e**.

**Figure S.39**: ^13^C NMR spectrum of compound **3f**.

**Figure S.40**: ^1^H NMR spectrum of compound **3g**.

**Figure S.41**: ^13^C NMR spectrum of compound **3g**.

**Figure S.42**: ^1^H NMR spectrum of compound **3h**.

**Figure S.43**: ^13^C NMR spectrum of compound **3h**.

**Figure S.44**: ^1^H NMR spectrum of compound **5a**.

**Figure S.45**: ^13^C NMR spectrum of compound **5a**.

**Figure S.46**: ^1^H NMR spectrum of compound **5b**.

**Figure S.47**: ^13^C NMR spectrum of compound **5b**.

**Figure S.48**: ^1^H NMR spectrum of compound **5c**.

**Figure S.49**: ^13^C NMR spectrum of compound **5c**.

**Figure S.50**: ^1^H NMR spectrum of compound **5d**.

**Figure S.51**: ^13^C NMR spectrum of compound **5d**.

**Figure S.52**: ^1^H NMR spectrum of compound **5e**.

**Figure S.53**: ^13^C NMR spectrum of compound **5e**.

**Figure S.54**: ^1^H NMR spectrum of compound **5f**.

**Figure S.55**: ^13^C NMR spectrum of compound **5f**.

**Figure S.56**: ^1^H NMR spectrum of compound **4a**.

**Figure S.57**: ^13^C NMR spectrum of compound **4a**.

**Figure S.58**: ^1^H NMR spectrum of compound **4b**.

**Figure S.59**: ^13^C NMR spectrum of compound **4b**.

**Figure S.60**: ^1^H NMR spectrum of compound **4c**.

**Figure S.61**: ^13^C NMR spectrum of compound **4c**.

**Figure S.62**: ^1^H NMR spectrum of compound **4d**.

**Figure S.63**: ^13^C NMR spectrum of compound **4d**.

**Figure S.64**: ^1^H NMR spectrum of compound **4e**.

**Figure S.65**: ^13^C NMR spectrum of compound **4e**.

**Figure S.66**: ^1^H NMR spectrum of compound **4f**.

**Figure S.67**: ^13^C NMR spectrum of compound **4f**.

**Figure S.68**: ^1^H NMR spectrum of compound **4g**.

**Figure S.69**: ^13^C NMR spectrum of compound **4g**.

**Figure S.70**: ^1^H NMR spectrum of compound **4h**.

**Figure S.71**: ^13^C NMR spectrum of compound **4h**.

**Figure S.72**: ^1^H NMR spectrum of compound **7a**.

**Figure S.73**: ^13^C NMR spectrum of compound **7a**.

**Figure S.74**: ^1^H NMR spectrum of compound **7b**.

**Figure S.75**: ^13^C NMR spectrum of compound **7b**.

**Figure S.76**: ^1^H NMR spectrum of compound **7c**.

**Figure S.77**: ^13^C NMR spectrum of compound **7c**.

**Figure S.78**: ^1^H NMR spectrum of compound **7d**.

**Figure S.79**: ^13^C NMR spectrum of compound **7d**.

**Figure S.80**: ^1^H NMR spectrum of compound **7e**.

**Figure S.81**: ^13^C NMR spectrum of compound **7e**.

**Figure S.82**: ^1^H NMR spectrum of compound **7f**.

**Figure S.83**: ^13^C NMR spectrum of compound **7f**.

**HRMS-ESI charts of compounds 2a-h, 4a-h, 5a-f and 7a-f**

**Figure S.84**: HRMS-ESI chart of compound **2a**.

**Figure S.85**: HRMS-ESI chart of compound **2c**.

**Figure S.86**: HRMS-ESI chart of compound **2d**.

**Figure S.87**: HRMS-ESI chart of compound **2e**.

**Figure S.88**: HRMS-ESI chart of compound **2g**.

**Figure S.89**: HRMS-ESI chart of compound **2h**.

**Figure S.90**: HRMS-ESI chart of compound **5a.**

**Figure S.91**: HRMS-ESI chart of compound **5b.**

**Figure S.92**: HRMS-ESI chart of compound **5c.**

**Figure S.93**: HRMS-ESI chart of compound **5d.**

**Figure S.94**: HRMS-ESI chart of compound **5e.**

**Figure S.95**: HRMS-ESI chart of compound **5f.**

**Figure S.96**: HRMS-ESI chart of compound **4a.**

**Figure S.97**: HRMS-ESI chart of compound **4b.**

**Figure S.98**: HRMS-ESI chart of compound **4c.**

**Figure S.99**: HRMS-ESI chart of compound **4d.**

**Figure S.100**: HRMS-ESI chart of compound **4e.**

**Figure S.101**: HRMS-ESI chart of compound **4f.**

**Figure S.102**: HRMS-ESI chart of compound **4g.**

**Figure S.103**: HRMS-ESI chart of compound **4h.**

**Figure S.104**: HRMS-ESI chart of compound **7a.**

**Figure S.105**: HRMS-ESI chart of compound **7b.**

**Figure S.106**: HRMS-ESI chart of compound **7c.**

**Figure S.107**: HRMS-ESI chart of compound **7d.**

**Figure S.108**: HRMS-ESI chart of compound **7e.**

**Figure S.109**: HRMS-ESI chart of compound **7f.**

**Figure S.110**: Microanalyses results.

**Chromatographic Conditions for HPLC:**

Analysis was carried out using Agilent 1100 series apparatus. Detection method was using diode array (DAD) ultraviolet (UV) detector was used at 254 nm. Column used was C18 column Zorbax ODS (4.6 x 150 mm i.d.,5 mm) and eluent was acetonitrile: methanol (50:50), flow rate = 1 mL/min, temperature 25 °C.

**Figure S.111**: HPLC Chromatogram for compound **4c**.

**Figure S.112**: HPLC Chromatogram for compound **7a**.

**Dose response curves of the target compounds on COX isoenzymes and 5-LOX**

Compound 4a

| Concentration (Log) | % Inhibition |
| --- | --- |
| 2 | 88.6 |
| 1 | 58 |
| 0 | 46.4 |
| -1 | 29.5 |
| -2 | 2.3 |

**Figure S.113**: Dose response curve of compound **4a** on COX-1.

Compound 4b

| Concentration (Log) | % Inhibition |
| --- | --- |
| 2 | 77.7 |
| 1 | 42.4 |
| 0 | 11.5 |
| -1 | 5.53 |
| -2 | 1.96 |

**Fig. S.114:** Dose response curve of compound **4b** on COX-1.

Compound 4c

| Concentration (Log) | % Inhibition |
| --- | --- |
| 2 | 81.5 |
| 1 | 54.4 |
| 0 | 14.2 |
| -1 | 6.18 |
| -2 | 1.78 |

**Fig. S.115:** Dose response curve of compound **4c** on COX-1.

Compound 4d

| Concentration (Log) | % Inhibition |
| --- | --- |
| 2 | 70.6 |
| 1 | 24.5 |
| 0 | 5.47 |
| -1 | 2.14 |
| -2 | 0.34 |

**Fig. S.116:** Dose response curve of compound **4d** on COX-1.

Compound 4e

| Concentration (Log) | % Inhibition |
| --- | --- |
| 2 | 75 |
| 1 | 27.7 |
| 0 | 10.4 |
| -1 | 5.45 |
| -2 | 0.54 |

**Fig. S.117:** Dose response curve of compound **4e** on COX-1.

Compound 4f

| Concentration (Log) | % Inhibition |
| --- | --- |
| 2 | 88 |
| 1 | 64.4 |
| 0 | 33.5 |
| -1 | 10.5 |
| -2 | 4.18 |

**Fig. S.118:** Dose response curve of compound **4f** on COX-1.

Compound 4g

| Concentration (Log) | % Inhibition |
| --- | --- |
| 2 | 77.1 |
| 1 | 23.1 |
| 0 | 7.55 |
| -1 | 2.47 |
| -2 | 0.66 |

**Fig. S.119:** Dose response curve of compound **4g** on COX-1.

Compound 7a

| Concentration (Log) | % Inhibition |
| --- | --- |
| 2 | 80.2 |
| 1 | 46.3 |
| 0 | 19 |
| -1 | 6.14 |
| -2 | 2.25 |

**Fig. S.120:** Dose response curve of compound **7a** on COX-1.

Compound 7b

| Concentration (Log) | % Inhibition |
| --- | --- |
| 2 | 90 |
| 1 | 63.2 |
| 0 | 30.7 |
| -1 | 8.22 |
| -2 | 1.87 |

**Fig. S.121:** Dose response curve of compound **7b** on COX-1.

Compound 7c

| Concentration (Log) | % Inhibition |
| --- | --- |
| 2 | 72 |
| 1 | 29.3 |
| 0 | 7.75 |
| -1 | 2.75 |
| -2 | 0.32 |

**Fig. S.122:** Dose response curve of compound **7c** on COX-1.

Compound 7d

| Concentration (Log) | % Inhibition |
| --- | --- |
| 2 | 63.1 |
| 1 | 12.9 |
| 0 | 3.09 |
| -1 | 0.7 |
| -2 | 0.3 |

**Fig. S.123:** Dose response curve of compound **7d** on COX-1.

Compound 7e

| Concentration (Log) | % Inhibition |
| --- | --- |
| 2 | 74.7 |
| 1 | 26.5 |
| 0 | 11.5 |
| -1 | 4 |
| -2 | 0.53 |

**Fig. S.124:** Dose response curve of compound **7e** on COX-1.

Compound 7f

| Concentration (Log) | % Inhibition |
| --- | --- |
| 2 | 77.1 |
| 1 | 41.4 |
| 0 | 12 |
| -1 | 6.21 |
| -2 | 1.09 |

**Fig. S.125:** Dose response curve of compound **7f** on COX-1.

Compound 4a

| Concentration (Log) | % Inhibition |
| --- | --- |
| 2 | 83.6 |
| 1 | 60.4 |
| 0 | 34.9 |
| -1 | 15.9 |
| -2 | 6.08 |

**Fig. S.126:** Dose response curve of compound **4a** on COX-2.

Compound 4b

| Concentration (Log) | % Inhibition |
| --- | --- |
| 2 | 76.7 |
| 1 | 49 |
| 0 | 14.8 |
| -1 | 7.44 |
| -2 | 2.48 |

**Fig. S.127:** Dose response curve of compound **4b** on COX-2.

Compound 4c

| Concentration (Log) | % Inhibition |
| --- | --- |
| 2 | 92.7 |
| 1 | 76.6 |
| 0 | 40 |
| -1 | 27.6 |
| -2 | 13.2 |

**Fig. S.128:** Dose response curve of compound **4c** on COX-2.

Compound 4d

| Concentration (Log) | % Inhibition |
| --- | --- |
| 2 | 74.4 |
| 1 | 24.2 |
| 0 | 9.69 |
| -1 | 6.15 |
| -2 | 1.45 |

**Fig. S.129:** Dose response curve of compound **4d** on COX-2.

Compound 4e

| Concentration (Log) | % Inhibition |
| --- | --- |
| 2 | 79.9 |
| 1 | 44.6 |
| 0 | 15.8 |
| -1 | 4.11 |
| -2 | 2.3 |

**Fig. S130:** Dose response curve of compound **4e** on COX-2.

Compound 4f

| Concentration (Log) | % Inhibition |
| --- | --- |
| 2 | 85.3 |
| 1 | 55.4 |
| 0 | 35.2 |
| -1 | 14.3 |
| -2 | 6.29 |

**Fig. S.131:** Dose response curve of compound **4f** on COX-2.

Compound 4g

| Concentration (Log) | % Inhibition |
| --- | --- |
| 2 | 73.5 |
| 1 | 44 |
| 0 | 16.6 |
| -1 | 7.79 |
| -2 | 4.24 |

**Fig. S.132:** Dose response curve of compound **4g** on COX-2.

Compound 4h

| Concentration (Log) | % Inhibition |
| --- | --- |
| 2 | 66.5 |
| 1 | 40.9 |
| 0 | 11.8 |
| -1 | 5.57 |
| -2 | 2 |

**Fig. S.133:** Dose response curve of compound **4h** on COX-2.

Compound 7a

| Concentration (Log) | % Inhibition |
| --- | --- |
| 2 | 90.2 |
| 1 | 62.5 |
| 0 | 40 |
| -1 | 22.8 |
| -2 | 11.8 |

**Fig. S.134:** Dose response curve of compound **7a** on COX-2.

Compound 7b

| Concentration (Log) | % Inhibition |
| --- | --- |
| 2 | 86.4 |
| 1 | 64.8 |
| 0 | 39.5 |
| -1 | 22 |
| -2 | 5.07 |

**Fig. S.135:** Dose response curve of compound **7b** on COX-2.

Compound 7c

| Concentration (Log) | % Inhibition |
| --- | --- |
| 2 | 73 |
| 1 | 36.3 |
| 0 | 14.5 |
| -1 | 8.51 |
| -2 | 3.09 |

**Fig. S.136:** Dose response curve of compound **7c** on COX-2.

Compound 7d

| Concentration (Log) | % Inhibition |
| --- | --- |
| 2 | 56.2 |
| 1 | 26.7 |
| 0 | 9.93 |
| -1 | 1.77 |
| -2 | 0.2 |

**Fig. S.137:** Dose response curve of compound **7d** on COX-2.

Compound 7e

| Concentration (Log) | % Inhibition |
| --- | --- |
| 2 | 67 |
| 1 | 40.3 |
| 0 | 16.9 |
| -1 | 8.95 |
| -2 | 3.79 |

**Fig. S.138:** Dose response curve of compound **7e** on COX-2.

Compound 7f

| Concentration (Log) | % Inhibition |
| --- | --- |
| 2 | 76.6 |
| 1 | 55.7 |
| 0 | 24.2 |
| -1 | 14.2 |
| -2 | 7.53 |

**Fig. S.139:** Dose response curve of compound **7f** on COX-2.

Compound 4a

| Concentration (Log) | % Inhibition |
| --- | --- |
| 2 | 86.8 |
| 1 | 68.6 |
| 0 | 36.1 |
| -1 | 14.5 |
| -2 | 3.41 |

**Fig. S.140:** Dose response curve of compound **4a** on 5-LOX.

Compound 4b

| Concentration (Log) | % Inhibition |
| --- | --- |
| 2 | 81.6 |
| 1 | 51.8 |
| 0 | 18.3 |
| -1 | 8.4 |
| -2 | 1.94 |

**Fig. S.141:** Dose response curve of compound **4b** on 5-LOX.

Compound 4c

| Concentration (Log) | % Inhibition |
| --- | --- |
| 2 | 95.1 |
| 1 | 85.3 |
| 0 | 54.5 |
| -1 | 33.3 |
| -2 | 19.7 |

**Fig. S.142:** Dose response curve of compound **4c** on 5-LOX.

Compound 4d

| Concentration (Log) | % Inhibition |
| --- | --- |
| 2 | 80 |
| 1 | 45.7 |
| 0 | 13.4 |
| -1 | 4.96 |
| -2 | 1.25 |

**Fig. S.143:** Dose response curve of compound **4d** on 5-LOX.

Compound 4e

| Concentration (Log) | % Inhibition |
| --- | --- |
| 2 | 77 |
| 1 | 26.1 |
| 0 | 13 |
| -1 | 4.14 |
| -2 | 0.91 |

**Fig. S.144:** Dose response curve of compound **4e** on 5-LOX.

Compound 4f

| Concentration (Log) | % Inhibition |
| --- | --- |
| 2 | 94.4 |
| 1 | 79.8 |
| 0 | 51.4 |
| -1 | 32.6 |
| -2 | 14.4 |

**Fig. S.145:** Dose response curve of compound **4f** on 5-LOX.

Compound 4g

| Concentration (Log) | % Inhibition |
| --- | --- |
| 2 | 78.8 |
| 1 | 44.6 |
| 0 | 20.9 |
| -1 | 6.14 |
| -2 | 1.97 |

**Fig. S.146:** Dose response curve of compound **4g** on 5-LOX.

Compound 4h

| Concentration (Log) | % Inhibition |
| --- | --- |
| 2 | 77 |
| 1 | 38.8 |
| 0 | 15.6 |
| -1 | 8.92 |
| -2 | 1.42 |

**Fig. S.147:** Dose response curve of compound **4h** on 5-LOX.

Compound 7a

| Concentration (Log) | % Inhibition |
| --- | --- |
| 2 | 92.1 |
| 1 | 78.3 |
| 0 | 51.2 |
| -1 | 21.2 |
| -2 | 9.17 |

**Fig. S.148:** Dose response curve of compound **7a** on 5-LOX.

Compound 7b

| Concentration (Log) | % Inhibition |
| --- | --- |
| 2 | 92.6 |
| 1 | 80.6 |
| 0 | 43.5 |
| -1 | 12.6 |
| -2 | 3.5 |

**Fig. S.149:** Dose response curve of compound **7b** on 5-LOX.

Compound 7c

| Concentration (Log) | % Inhibition |
| --- | --- |
| 2 | 75.1 |
| 1 | 35.6 |
| 0 | 17.6 |
| -1 | 7.69 |
| -2 | 1.29 |

**Fig. S.150:** Dose response curve of compound **7c** on 5-LOX.

Compound 7d

| Concentration (Log) | % Inhibition |
| --- | --- |
| 2 | 70.5 |
| 1 | 25.9 |
| 0 | 4.33 |
| -1 | 1.5 |
| -2 | 0.72 |

**Fig. S.151:** Dose response curve of compound **7d** on 5-LOX.

Compound 7e

| Concentration (Log) | % Inhibition |
| --- | --- |
| 2 | 71.6 |
| 1 | 36.3 |
| 0 | 15 |
| -1 | 4.33 |
| -2 | 0.92 |

**Fig. S.152:** Dose response curve of compound **7e** on 5-LOX.

Compound 7f

| Concentration (Log) | % Inhibition |
| --- | --- |
| 2 | 93.9 |
| 1 | 80.6 |
| 0 | 53.3 |
| -1 | 26.3 |
| -2 | 9.94 |

**Fig. S.153:** Dose response curve of compound **7f** on 5-LOX.
